# Effects of reduced nitrogen on the nifH-harboring soil microbiome in a soybean-maize strip intercropping system

**DOI:** 10.3389/fmicb.2026.1770580

**Published:** 2026-03-10

**Authors:** Fang Liu, Lisong Shi, Shuo Yan, Yiling Zhang, Mengxi Zhang, Tonghao Han, Xuan Zhao, Zhanjun Li, Ning Niu

**Affiliations:** 1 Shijiazhuang Academy of Agricultural and Forestry Sciences, Shijiazhuang, China; 2 Hebei Soybean Institute of Applied Technology, Shijiazhuang, China

**Keywords:** soybean-maize relay strip intercropping, *nifH* gene, rhizosphere microbes, soil microbial diversity, N-fixing bacteria

## Abstract

**Introduction:**

Nitrogen (N) is a core limiting factor for crop growth, with approximately 50% of global food production relying on chemical N fertilizer inputs. However, excessive N application results in N use efficiency below 40%, and unabsorbed N triggers environmental problems. Maize-soybean relay strip intercropping (MSSI) enhances vertical resource partitioning, increases land productivity, and optimizes N utilization, but its effects on *nifH*-marked N-fixing microbiota under reduced N input remain unclear. This study aimed to investigate the abundance and diversity of N-fixing microbiota in response to the MSSI system with reduced N application.

**Methods:**

A 2-year field experiment was conducted in two soil textures (sandy loam in Wuji and medium loam in Gaocheng) including three cropping systems: monocropping maize, monocropping soybean, and MSSI. To further explore the underlying mechanism, an N gradient experiment with four fertilizer rates was established in Wuji. At the maturation stage, rhizosphere soil samples were collected, and q-PCR, enzyme activity assays, and high-throughput sequencing were used to analyze N cycle-related marker genes, enzyme activities, and *nifH* gene abundance and diversity.

**Results:**

The MSSI system maintained maize yields comparable to monocropped maize, while soybean yields reached 60.1–69.6% of monocropped levels. MSSI significantly increased *nifH* gene abundance in soybean rhizosphere soil, but reduced the Chao1, Shannon, Simpson, and observed species indices of N-fixing microbiota. Specifically, MSSI decreased N-fixer diversity (Shannon: −18.2%) and richness (Chao1: −12.5%), whereas the 25% reduced N input treatment (ISN_25_) enhanced diversity (Shannon: +15.7%) by improving community evenness without altering species richness.

**Discussion:**

Our results demonstrate that the MSSI system significantly alters soil N fertility and the community structure of *nifH*-marked N-fixing bacteria. The reduced N input combined with MSSI can optimize N utilization by regulating N-fixing microbial communities, providing a theoretical basis for sustainable agricultural practices that balance food security and ecological protection.

## 1 Introduction

Since the green revolution has increased crop yields through the wide application of the semidwarfing revolution for plant cultivars, the use of pesticides and extensive use of mineral fertilizers, the availability of nitrogen (N) in the soil has been a major limiting factor for crops. N, as a key element of proteins, is an essential nutrient for crops. Soil N cycling affects the productivity and sustainability of soil ecosystems. However, excessive application of N fertilizer can damage the soil environment and affect the soil microecology on farm. In recent years, scientists have begun to pay attention to the application of soil N fertilizer transformation and circulation.

Biological N fixation in root nodules is a primary N source for *legumes*, whereby diazotrophs use nitrogenase to convert atmospheric nitrogen (N_2_) into ammonium (NH_4_^+^), the bioavailable form of N directly utilized by plants (Aasfar et al., 2021). The ammonium ions obtained by biological N-fixation can be absorbed by plant roots, thus providing additional N sources for the ecosystem, which is one of the main routes of material circulation in farmland ecosystems. Soil microorganisms play a very important role in soil N cycling. Soil microorganisms play an important role in promoting N nutrient cycling, decomposition and transformation of soil organic matter (Jin et al., 2024; Navarro et al., 2025). An increasing number of studies have identified N-fixing bacteria as key players in the N cycle, while denitrifying microorganisms drive the return of reactive nitrogen to the atmosphere. The genes encoding nitrogenases (including *nifH*, *nifK*, and *nifD*) are highly conserved in different N-fixing organisms. The *nifH* gene, encoding the reductase component of nitrogenase, is well conserved and has extensive data. In phylogenetic analysis, the *nifH* gene exhibits variable regions and phylogenetic topology to comparable to those of the 16S rRNA gene, thus serving as a core molecular marker for profiling N-fixing microbial communities and assessing diversity (Hao et al., 2022; Yang et al., 2024). Soil N-fixing microorganisms are an important part of the rhizosphere soil environment, and community diversity can reflect the metabolic patterns and physiological functions of soil microecology. The abundance and community composition of N-fixing bacteria are crucial for soil N-fixation and the balance of the N cycle, serving as a significant indicator of soil quality (Li and Cupples, 2021). Therefore, studying the difference in the community structure of soil N-fixing bacteria under different N fertilizer application rates or different planting patterns is highly important for the establishment of reasonable planting patterns and soil N recycling (Zheng et al., 2022). Many studies have shown that the composition of N-fixing bacterial communities is regulated by abiotic and biological factors, such as vegetation type, tillage method, and soil physicochemical properties (pH, available N).

At present, more than 60 genera of N-fixing microorganisms have been identified, among which *Desulfovibrio* belongs to the phylum *Pseudomondota* (Wewalwela et al., 2021). Therefore, the *nifH* gene was selected to study the phylogeny, diversity and abundance of N-fixing bacteria and provides a useful pathway for studying the distribution and diversity of N-fixing bacteria (Zhang C. et al., 2021; Zhang Y. et al., 2021).

Intercropping, the practice of cocultivation two or more crop species in the same field, enhances agricultural sustainability by leveraging species synergies through crop diversity (Maitra et al., 2023). On the basis of the individual crop yields of the component species, this agricultural diversification technique enables decreasing inputs while attaining larger crop yields than anticipated. This strategy uses facilitative and competitive interactions to increase crop yield and N use efficiency (Chai et al., 2021).

Generally, the N accumulation and yield of soybean increase with N fertilizer application (Yao et al., 2017). The MSSI system uniquely balances high yields (with a land equivalent ratio of 1.14–1.50) and reduces N inputs (20–30% less than monocropping) by increasing N use efficiency (13.6–129.1%) (Li et al., 2024; Zhang et al., 2025). High N use efficiency mitigates environmental N losses and enhances agricultural sustainability (Zhang C. et al., 2021; Zhang Y. et al., 2021). Intercropping with soybean improved biological nitrogen fixation (BNF) and N utilization by altering the composition of the soil bacterial community. However, the regulatory mechanisms underlying the effects of reduced N fertilizer application on the structure and function of the *nifH*-marked N-fixing microbiota in the soybean rhizosphere are still unclear.

In recent years, it has been suggested that the intercropping of *legumes*, which are a composite population of various crop species, has the potential to optimize N utilization in *legume*-based intercropping systems (Yao et al., 2024; Yang et al., 2023). This study investigated three planting patterns under four levels of N fertilizer application: monocropping soybean (MS), maize/soybean relay strip intercropping (MSSI), and monocropping maize (MM), each of which was subjected to four N fertilizer application (N_0_, N_25_, N_50_, and N_100_). To investigate potential changes in N fertilizer use and cyclic utilization in response to different cropping patterns, we conducted a quantitative real-time PCR analysis of the abundance of four functional genes that represent four critical steps in the soil N cycle. These genes include ammonium monooxygenase of bacteria (*amoA*) for nitrification, nitrite reductase (*nirK*) for denitrification, the key enzyme (*nxrA*) for the oxidation of NO_2_^−^ to NO_3_^−^ in nitrite-oxidizing bacteria, and a subunit of nitrogenase (*nifH*) for N_2_ assimilation. We analyzed the correlation of *nifH* abundance and diversity with soil physicochemical properties and soil enzyme activity, which provides a theoretical foundation for further elucidation of the mechanism of improvement in N-reduction and efficiency in maize-soybean relay strip intercropping system.

## 2 Materials and methods

### 2.1 Experimental site and design

The field experiments were established in the Wuji (WJ) and Gaocheng (GC) Countries of Shijiazhuang city, Hebei Province, China (115°02′E–38°13′N, 114°43′–37°96), in 2023–2024. These regions are characterized by a temperate continental monsoon climate. The average annual temperature is 12.94–13.04 °C. The average precipitation in WJ during the soybean and maize growth periods was 37.5 mm in June, 288.9 mm in July, 184.4 mm in August, 51.5 mm in September and 3.3 mm in October. The average precipitation in the GC during the soybean and maize growth periods was 32.9 mm in June, 219.2 mm in July, 137.6 mm in August, 70.2 mm in September and 80.0 mm in October. The tested soil was classified into two types: medium loam soil in GC and sandy loam soil in WJ. The general chemical properties of the soil are shown in Table 1.

**Table 1:** General chemical properties of soil.

Experiment site	Soil texture	TN (g kg^−1^)	OM (g kg^−1^)	AN (g kg^−1^)	AP (g kg^−1^)	AK (g kg^−1^)	pH
GC	MLS	1.50 ± 0.03	26.49 ± 0.76	132.13 ± 3.54	59.33 ± 1.25	187.33 ± 11.22	7.09 ± 0.04
WJ	SLS	1.62 ± 0.02	28.85 ± 1.21	145.95 ± 10.21	54.23 ± 6.25	187.50 ± 14.68	7.33 ± 0.05

TN, total nitrogen; OM, organic matter; AN, alkaline hydrolysis N; AK, available K; AP, available P; MLS, medium loam soil; SLS, sandy loam soil; GC, the field experiments country Gaocheng; WJ, the field experiments country Wuji. Data in this table are represented by mean ± standard deviation (n = 3).

The soybean variety used for testing was “Shi 936,” which was supplied by the Shijiazhuang Academy of Agricultural and Forestry Sciences, and the maize variety was “Weike 908,” which was supplied by the Zhengzhou Weike Crop Breeding Company. Shi 936 is a soybean variety suitable for the MSSI system, and has the characteristics of shade tolerance, stable yield and high yield.

This study was conducted in a wheat-maize/soybean rotation system. Wheat was planted in October and harvested the following June, followed by direct seeding of maize or soybean from June to October. To compare the distribution of N-fixing bacteria between the MSSI and monocropping cropping patterns, a split plot design with two factors, i.e., the planting pattern and nitrogenous fertilizer was used. The main plot was planted with three cropping systems: monocropping maize (MM), the MSSI system (strip intercropping system with 4 rows of soybean and 2 rows of maize), and monocropping soybean (MS), and the total amount of N fertilizers was applied to the secondary plot.

**Figure 1:**
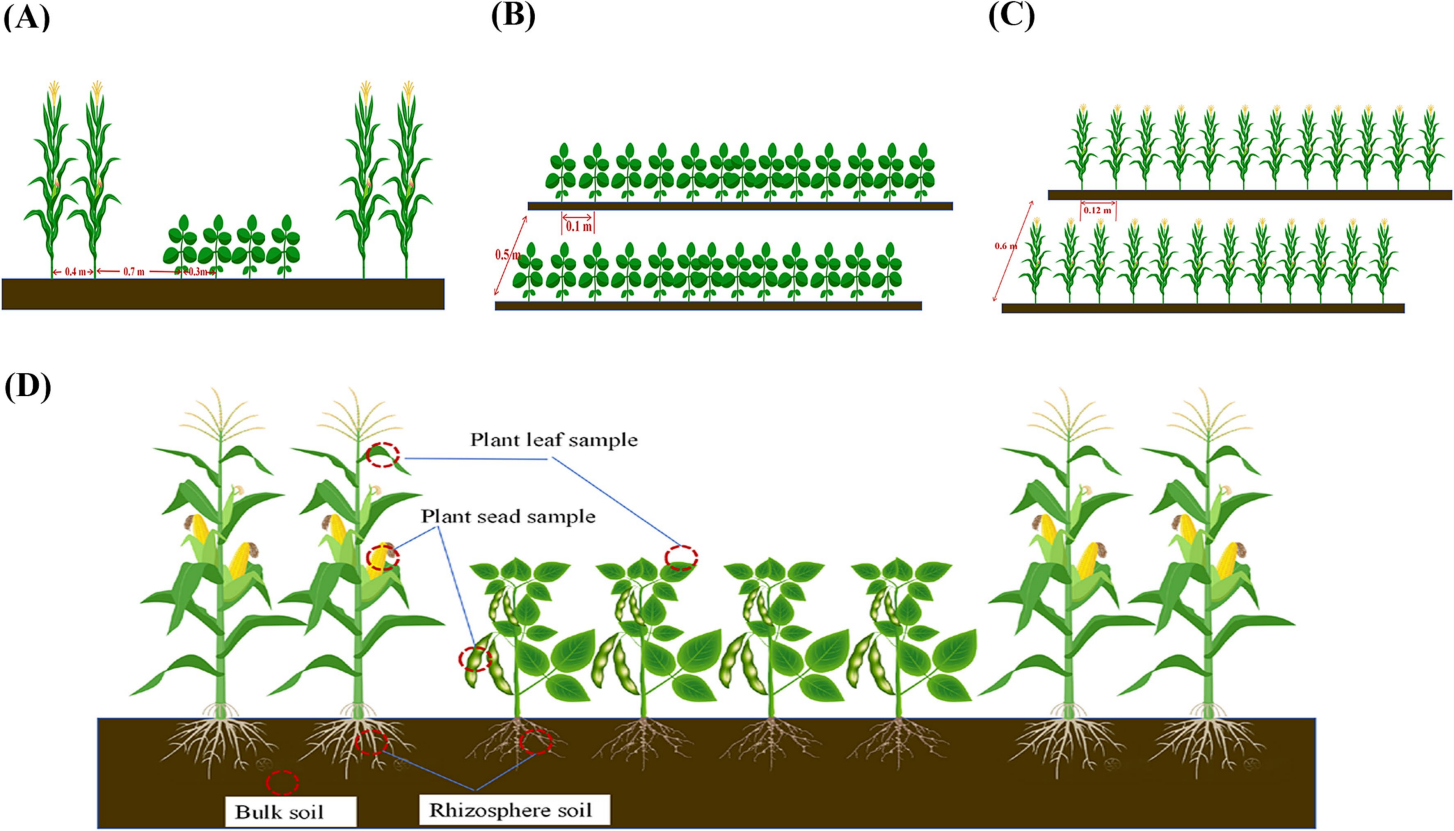
Schematic representation of field experimental setups in this study. **(A)** Distribution of maize-soybean relay strip intercropping system trial plots, 0.7 m refers to the spacing between soybean and maize, while 0.4 m or 0.3 m is the spacing between maize or soybean within a given row. **(B)** Distribution of monocropping soybean trial plots, 0.5 m refers to the spacing between rows, while 0.1 m is the spacing between soybean. **(C)** Distribution of monocropping maize trial plots; 0.6 m refers to the spacing between rows, while 0.12 m is the spacing between maize. **(D)** The red circles represent where the plants and soil were sampled.

The MSSI field was characterized as alternating strips of maize and soybean, where two rows of maize were arranged adjacent to four rows of soybean, forming a repeating intercropping unit (Figure 1A). For the soybean strips, the interrow distance was 0.30 m, with a within-row spacing of 0.10 m. For the maize strips, the within-row plant spacing was 0.12 m, and the interrow distance was 0.40 m. The isolation distance between the outermost row of maize strips and the outermost row of adjacent soybean strips was 0.70 m. A monocropping of maize was planted with 0.60 m line spacing and 0.12 m row spacing (Figure 1B). The line spacing for the soybean monocropping system was 0.50 m, whereas the row spacing was 0.10 m (Figure 1C).

To further analyze the microecological effects of N fertilizer application, we established four N gradients in the 2024 in the WJ experiment: N_100_ (100% conventional N, 150 kg hm^−2^), N_50_ (50% reduction, 75 kg hm^−2^), N_25_ (75% reduction, 37.5 kg hm^−2^), and N_0_ (0 kg hm^−2^). Each treatment included three biological replicates, with a plot area of 18 m^2^ (Supplementary Table S1).

Uniform broadcast application was used in N fertilizer arrangement with stage-specific adjustments. In the first stage (2 year, 2 site) experiment, urea was evenly spread at a consistent rate of 150 kg hm^−2^ across all plots and incorporated into 0–20 cm soil. In the second stage (N gradient) experiment, plot-specific urea dosages matching different N levels were uniformly broadcast using the same tillage method, ensuring only N supply varied.

### 2.2 Soil sampling, biomass, and soil enzyme activity analysis

On September 26, 2024, 48 rhizosphere soil samples were collected from 12 different test districts. To acquire the rhizosphere soil, soybean roots were excavated to 0.2 m depth, and maize roots were excavated to 0.5 m below the ground surface via shovels. The soil was separated from the roots of each plant. Soil loosely attached to the root surface is carefully shaken or brushed off as plant rhizosphere soil (Figure 1D).

The mono-rhizosphere soil samples (MS) were collected from soybean roots, whereas the interrhizosphere soil samples (IS) were collected from the root zones of the maize/soybean intercropping systems. Accordingly, bulk soil was collected outside the rhizosphere zone. The composite samples were homogenized and separated, and visible roots, branches, and stones were removed. In the end, a total of 48 samples were collected and promptly transferred to the laboratory. Once present, they were maintained at −20 °C for the purpose of DNA extraction as well as the physical and chemical analysis.

The pH of the soil was measured via a pH meter at a water-to-soil ratio of 2.5:1 (volume to weight) (Luo et al., 2017). We used a volumetric approach to quantify the amount of soil organic matter (SOM). Total N (TN) in the soil was determined via the micro-Kjeldahl technique. In accordance with the alkali-hydrolysis diffusion method, the available N (AN) in the soil was measured (Vance et al., 1987), and the activities of soil urease (Yuan et al., 2006), hydroxylamine reductase, nitrate reductase, and nitrite reductase were measured according to Guan (2008).

### 2.3 DNA extraction and quantification of gene copies number for *nifH*, *nxrA*, *nirK*, and *amo*A

Following the manufacturer’s recommendations, 0.5 g of soil was used to extract soil DNA via a potent DNA isolation kit (M5635-02, OmegaBio-Tek, Norcross, GA, United States). The amount and quality of DNA were measured with a NanoDrop NC2000 spectrophotometer (Thermo Fisher Scientific, Waltham, MA, United States) and 1% agarose gel electrophoresis was performed (Hallin and Lindgren, 1999; Poly et al., 2008; Rösch et al., 2002; Rotthauwe et al., 1997).

A real-time PCR system (Roche, Inc., United States) with primers was used to perform qPCR tests to determine the copy counts of the *nifH* (or *nxrA*, *nirK*, and *amoA*) genes (Laguerre et al., 2001). The diazotroph PCR mixture contained 2 μL of DNA template, 7.2 μL of sterilized Milli-Q water, 0.4 μL of 10 μM forward and reverse primers, and 10 μL of 2 × Real SYBR Mixture (Vazyme Nanjing, China). Two microliters of H_2_O template was used as the negative control in place of DNA in the reactions. Amplification process: First, the DNA was denatured at 95 °C for 5 min. The mixture was subsequently heated at 60 °C for 30 s, extended at 72 °C for 30 s, and finally elongated at 72 °C for 10 min. The plate was then read at 83 °C. Melting curve analysis and agarose gel electrophoresis were used in order to validate the specificity of the amplicon. The primers used in this experiment are listed in Supplementary Table S2.

### 2.4 Illumina MiSeq sequencing and bioinformatics analysis

PCR amplification of the *nifH* gene was performed via the forward primers PolyF (5′-TGCGAYCCSAARGCBGACTC-3′), and PolyR (5′-ATSGCCATCATYTCRCCGGA-3′) (Poly et al., 2001). The primers used for multiplex sequencing were modified to include sample-specific 7-bp barcodes. The PCR mixtures included 5 μL of Q5 reaction buffer (5×), 5 μL of Q5 high-fidelity GC buffer (5×), 0.25 μL of Q5 high-fidelity DNA polymerase (5 U/μL), 2 μL (2.5 mM) of dNTPs, 1 μL (10 μM) of each forward and reverse primer, 2 μL of DNA template, and 8.75 μL of ddH_2_O. The process of thermal cycling included denaturation at 98 °C for 2 min, followed by 30 cycles of denaturation at 98 °C for 30 s, annealing at 59.5 °C for 30 s, and extension at 72 °C for 45 s, with a final extension of 5 min at 72 °C. PCR amplicons were measured via the PicoGreen dsDNA Assay Kit (Invitrogen, Carlsbad, CA, United States) after being purified via Agencourt AMPure Beads (Beckman Coulter, Indianapolis, IN).

Following the individual quantification stage, amplicons were combined in equal quantities and subjected to paired 250 bp sequencing at Shanghai Personal Biotechnology Co., Ltd. (Shanghai, China) via the Illumina NovaSeq platform and the 500-cycle NovaSeq 6000 SP Reagent Kit. QIIME2 2022.11 was used for microbiome bioinformatics, with minor adjustments made in accordance with official tutorials.^1^ In summary, the demux plugin was employed to demultiplex raw sequence data, which was subsequently followed by the cutadapt plugin to cut primers. The fastq_mergepairs, fastq_filter, and derep_fullength functions in the Vsearch plugin were subsequently employed to merge, quality filter, and dereplicate the sequences. The unique sequences were subsequently clustered at 98% (via cluster_size), and chimeras were removed (via uchime_denovo). Finally, OTU representative sequences and an OTU table were produced by reclustering the nonchimerase sequences at 97%. Nonsingleton amplicon sequence variations were aligned via MAFFT and used to generate a phylogenetic tree via FastTree2. Alpha-diversity metrics, which include Chao1 (Chao, 1984), observed species, Shannon (Shannon, 1948; Shannon et al., 1950), Simpson (Simpson, 1949), Pielou’s evenness (Pielou, 1966), and Good’s coverage (Good, 1953), as well as beta diversity metrics, which include the Jaccard distance and Bray–Curtis dissimilarity, were estimated with the help of the diversity plugin. The samples were rarefied to 783,700 sequences per sample. The classify-sklearn naïve Bayes taxonomy classifier in the feature-classifier plugin (Pittard et al., 2020) was used to assign taxonomy to OTUs in comparison to the NCBI database (Abarenkov et al., 2024).

### 2.5 Statistical analyzes

This research adopted an experimental design characterized by phased progression. The first stage focused on cropping patterns, soil types, and years, and three-way analysis of variance (ANOVA) was used for group difference analysis. The second stage, which was based on the optimal basic conditions identified in the first phase, further explored the regulatory effect of N fertilizer application level gradients via two-way analysis of variance (ANOVA). All experimental data are expressed as the mean ± standard deviation (SD). Prior to ANOVA, the Shapiro–Wilk test was used to assess data normality, and the Levene test was applied to evaluate homogeneity of variance. If the data failed to meet the assumptions of a normal distribution or equal variance, log-transformation transformation was performed followed by retesting. If the assumption remained unmet after transformation, nonparametric tests (e.g., the Kruskal-Wallis H test) were adopted for intergroup difference analysis. Pearson correlation analysis was conducted to examine the correlations between soil bacterial ecological diversity and soil physicochemical properties. GraphPad software was used for statistical analysis and figure generation, with statistical significance defined as *p* < 0.05. Pearson analysis was used to examine the correlations between the ecological variety of soil bacteria and the physical and chemical characteristics of the soil. Statistical significance was defined as *p* < 0.05.

## 3 Results

### 3.1 *nifH* gene abundance, yield, and plant height of soybean and maize under different cropping systems

**Figure 2:**
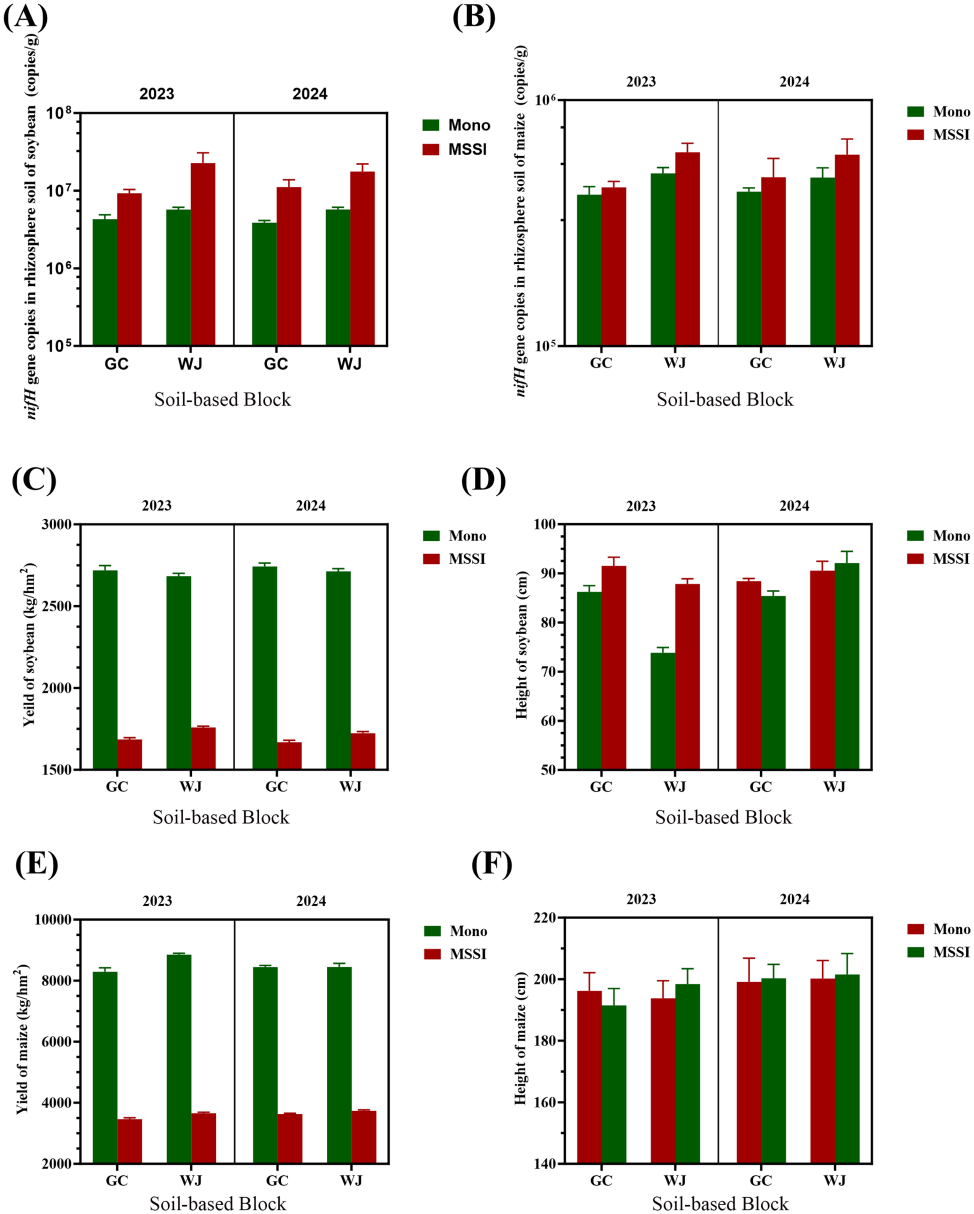
*nifH* gene abundance, yield, and plant height of soybean and maize under different cropping system. MSSI, maize-soybean relay strip intercropping system; Mono, monocropping system; GC, the field experiments country Gaocheng; WJ, the field experiments country Wuji. **(A)** *nifH* of soybean. **(B)** *nifH* of maize. **(C)** Yield of soybean. **(D)** Height of soybean. **(E)** Yield of maize. **(F)** Height of maize.

The effects of cropping patterns (Mono vs. MSSI), locations (GC vs. WJ), and years (2023 vs. 2024) on *nifH* gene copies, yield, and plant height of soybean and maize were analyzed (Figure 2). Three-way ANOVA (Supplementary Table S3) quantified the effects of soil property (S), year (Y), cropping pattern (C), and their interactions on soybean/maize nifH abundance, yield, and plant height. As illustrated in Figure 2, a consistent temporal and spatial pattern in nifH gene copy numbers was observed across all treatments and years, demonstrating high reproducibility of the experimental design. For soybean rhizosphere soil, MSSI significantly increased nifH gene copies compared to Mono in both locations and years, with higher values observed in 2024 than 2023 (Figure 2A). This may be due to improved resource partitioning, rhizosphere interaction, or microenvironmental modification resulting from the complementary architecture and physiological traits of the two species.

For maize, *nifH* gene copies in rhizosphere soil followed a similar trend: MSSI > Mono and 2024 > 2023 (Figure 2B). Notably, the *nifH* gene copies in soybean-associated rhizospheres were markedly higher than those in maize plots, with an average difference of approximately two orders of magnitude, indicating a significantly greater potential for biological N-fixation in the soybean rhizosphere regardless of cropping system. This is plausibly attributed to the symbiotic relationship between soybean (a leguminous plant) and diazotrophic bacteria such as *Bradyrhizobium* spp., which actively colonize root nodules and contribute substantially to nifH signal intensity.

For soybean *nifH*, cropping pattern (C) (*F* = 48.97, *p* = 0.0198) and the three-way interaction (S × C × Y) (*F* = 185.4, *p* = 0.0054) were significant, while soil property (S) had no effect. In contrast, maize *nifH* was driven by S (*F* = 813.6, *p* = 0.0012) and C (*F* = 53.87, *p* = 0.0181), with no interactive effects (Supplementary Table S3). Interestingly, the highest absolute abundance of the *nifH* gene was recorded in the soybean intercropping treatment situated in sandy loam soil, surpassing all other combinations. This observation implies that soil texture—particularly the favorable aeration, drainage, and root penetration characteristics of sandy loam—may synergistically enhance the establishment and function of N-fixing bacterial communities, especially under intercropping conditions.

The different cropping patterns and different N fertilizer applications signicantly influenced crops agronomic traits. Results from 2 years continuous field experiments across diverse soil types demonstrated that: within the MSSI cropping system, maize yield was comparable to that achieved under monocropping maize cultivation. However, soybean yield in the MSSI system was lower (about 60.8–65.6% of monocropping) than under mono soybean production. Soybean yield and plant height under MSSI were notably higher than Mono, with WJ showing better performance than GC across 2 years (Figures 2C,D). Soybean yield was strongly influenced by all factors: Y (*F* = 1764, *p* < 0.001), C (*F* = 483.5, *p* = 0.0021), and their interactions (e.g., S × C: *F* = 1895, *p* < 0.001). For maize yield, only S (*F* = 4,624, *p* < 0.001) and the three-way interaction (S × C × Y: *F* = 12.78, *p* = 0.0701) were significant, with C showing no effect (Supplementary Table S3).

These results highlight that cropping pattern is the primary driver of soybean traits and maize height, while soil property primarily regulates maize *nifH* and yield, with year effects concentrated in soybean yield. To further analyze the efficient mechanism of the MSSI model, we set up four N gradient field experiments at WJ.

### 3.2 Biological effects of MSSI on soybean under various N fertilizer applications

In monocropping (MS), soybean height and yield increased gradually with increasing N inputs, peaking at MSN₁₀₀ (92.1 cm, 2682.6 kg hm^−2^; Table 2). In contrast, in MSSI, these traits were maximized at ISN₂₅: height reached 91.5 cm, and yield (1722.7 kg hm^−2^) was significantly higher than other IS treatments (Table 2). Notably, ISN₂₅ also resulted in the greatest nodule number (41.5 per plant) in MSSI, which was substantially higher than MS treatments (max. 21.3 nodules; Supplementary Table S5).

**Table 2:** Growth traits of soybean in different treatments.

Sample ID	Height of soybean (cm)	Seeds number of soybean	Yield of soybean (kg hm^−2^)
MSN_0_	84.3 ± 1.4aA	94.4 ± 4.3aA	2236.7 ± 41.4aA
MSN_25_	86.2 ± 3.1abA	103.5 ± 5.1bA	2507.2 ± 53.5bA
MSN_50_	88.7 ± 2.6b	112.4 ± 2.9cA	2514.7 ± 17.8bA
MSN_100_	92.1 ± 3.5cA	125.8 ± 4.5d	2682.6 ± 23.6cA
ISN_0_	88.4 ± 1.9bB	89.3 ± 3.6aB	1591.6 ± 13.5aB
ISN_25_	91.5 ± 4.2cB	115.4 ± 4.0cB	1722.7 ± 13.8bB
ISN_50_	87.6 ± 2.8b	110.2 ± 2.7b	1725.1 ± 16.9bB
ISN_100_	73.8 ± 1.5aB	109.8 ± 3.4bB	1758.9 ± 10.3cB

MS, soybean in monocropping system; IS, soybean in MSSI system. The abbreviation N_0_, N_25_, N_50_, and N_100_ represent no N fertilizer, 75% N reduction, 50% N reduction and traditional N fertilizer, respectively. Data are mean ± SD (*n* = 3). Lowercase letters: differences among N levels in same cropping pattern (Tukey HSD test, *p* < 0.05); same letters = no significant difference. Uppercase letters: differences between Mono and MSSI groups at the same N level (independent samples *t*-test, *p* < 0.05); same letters = no significant difference.

Two-way ANOVA results (Supplementary Table S4) confirmed that cropping system (*F* ≥ 59.16, *p* < 0.001) and N rate (*F* ≥ 19.82, *p* < 0.001) strongly regulated soybean traits, with significant interactions for yield and nodule number (*F* ≥ 24.53, *p* < 0.001) (Supplementary Table S4). For quality traits, crude protein and fat contents showed no significant differences between Mono and MSSI systems (Supplementary Table S6), though N rate slightly modulated these indices. Collectively, these results indicate that a moderate N reduction (ISN₂₅) optimizes soybean growth, yield, and nodulation in MSSI, while MS requires full N input to achieve peak performance.

### 3.3 N content in soybean tissues under different cropping systems and N fertilization applications

**Figure 3:**
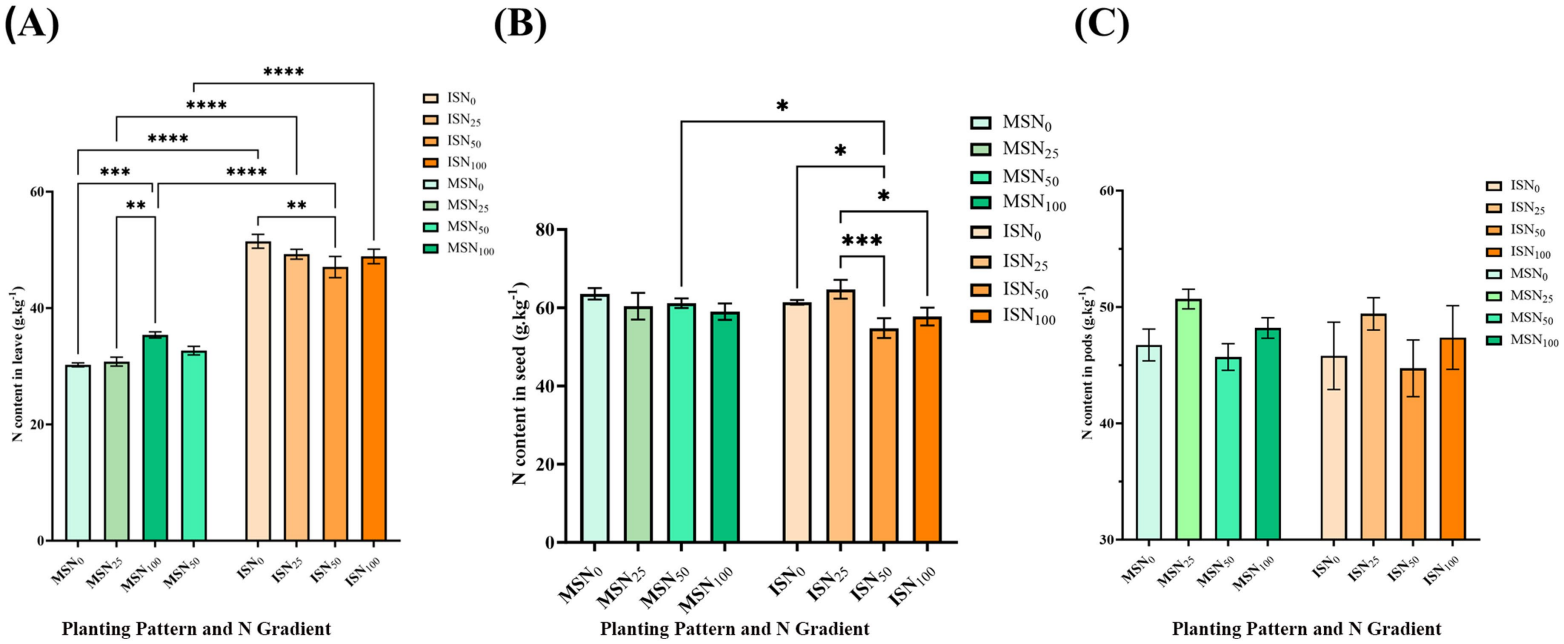
N content of soybean leaves, soybean pods and soybean seeds under different treatments. **(A)** N content of leaves. **(B)** N content of seeds. **(C)** N content of pods. MS, monocropping; IS, maize-soybean relay strip intercropping system. The abbreviation N_0_, N_25_, N_50_, and N_100_ represent no N fertilizer, 75% N reduction, 50% N reduction and traditional N fertilizer, respectively. Data are presented as means ± standard deviation (SD). ANOVA, Duncan test, * *p* < 0.05, ** *p* < 0.01, *** *p* < 0.001, **** *p* < 0.0001.

To further clarify the impacts of various planting patterns and N-reduction treatments on the yield of soybeans, we also determined the N contents of soybean leaves (6-leaf stage), mature pods, and harvested seeds (Figure 3). Two-way ANOVA results indicated a significant interaction between planting pattern and N rate on leaf N content (Figure 3A and Supplementary Table S4). The findings demonstrated that, at various N fertilizer application rates, the MSSI system may considerably increase the N content in soybean leaves by 60% as compared with that in monocropped soybean. In the soybean monocropping treatment, the N content in leaves increased slightly with increasing N fertilizer application, and the maximum N content was 51.47 g kg^−1^ under the ISN_0_ treatment (Figure 3A).

In contrast, N contents of pods and seeds were not significantly affected by either planting pattern or N fertilizer application levels (Figures 3B,C). These results indicate that the MSSI system can significantly increase the N content in the leaves of soybean plants during the growing period and that different cropping systems have little effect on soybean pods and seeds. Furthermore, various levels of N fertilizer application affected the yield of soybean (Table 2) but did not affect the N content of soybean or maize grains, thus N fertilizer application may have little effect on the protein accumulation and protein content of soybean (Supplementary Table S6).

### 3.4 MSSI effects on soybean rhizosphere soil properties

Two-way ANOVA results confirmed that planting pattern (monocropping vs. MSSI) and N rate (and their interaction) strongly regulated most rhizosphere soil properties (e.g., SOC: *F* = 224.10, *p* < 0.001; NO₃^−^-N: *F* = 347.70, *p* < 0.001), with only pH showing no significant variation between systems (Table 3; Supplementary Table S4). In low-N treatments (N₀), MSSI (ISN₀) significantly elevated rhizosphere SOC, SOM, TN, AN, and NO₃^−^-N relative to monocropping (MSN₀) (*p* < 0.05; Table 3; Supplementary Table S4), while NH₄^+^-N was 20.3% higher in ISN₀ (7.16 g kg^−1^) than MSN₀ (5.93 g kg^−1^). Under full N input (N₁₀₀), MSSI (ISN₁₀₀) had comparable TN, AN, SOC, and SOM to monocropping (MSN₁₀₀), but its NO₃^−^-N content (28.88 g kg^−1^) was 17.1% higher than MSN₁₀₀ (24.83 g kg^−1^; *p* < 0.05). In ISN₅₀, MSSI increased SOC and SOM by 26.8 and 26.4% (*p* < 0.05) compared to monocropping, while reducing NH₄^+^-N by 25.6% (*p* < 0.05). In ISN₂₅, MSSI maximized NO₃^−^-N (37.28 g kg^−1^, 2.3-fold higher than MSN₂₅) but decreased NH₄^+^-N to 4.43 g kg^−1^ (*p* < 0.05). Across treatments, rhizosphere TN and AN increased with N fertilizer application rate, and NO₃^−^-N in MSSI was consistently higher than in monocropping under the same N level (Table 4; Supplementary Table S4). These results indicate that MSSI reshapes soybean rhizosphere soil N composition (promoting NO₃^−^-N accumulation) and enhances organic matter content, especially under low-to-moderate N inputs.

**Table 3:** Number of OTUs and alpha diversity index of N-fixing bacteria at different sample sites.

Sample ID	No. of valid sequence	No. of OTUs	Goods-coverage	Shannon index	Simpson index	Observed_species	Chao 1 index
MSN_0_	102,932	228	0.99756a	0.7409b	0.131132b	289c	460c
MSN_25_	98,124	266	0.99683a	1.1696ab	0.229034a	340b	576
MSN_50_	95,515	388	0.99562a	1.3606a	0.257231a	461a	780a
MSN_100_	99,028	365	0.99582a	1.3653a	0.263376a	435a	753a
ISN_0_	106,135	230	0.99768a	0.7157b	0.129184b	270c	429c
ISN_25_	95,005	313	0.99661a	1.3605a	0.269459a	371b	630b
ISN_50_	98,012	232	0.99738a	0.8750b	0.158303b	304bc	485c
ISN_100_	88,949	278	0.99692a	1.0933ab	0.223649a	340b	559bc

MS, soybean in monocropping system; IS, soybean in MSSI system. The abbreviation N_0_, N_25_, N_50_, and N_100_ represent no N fertilizer, 75% N reduction, 50% N reduction and traditional N fertilizer, respectively. The numbers in the table are the mean value standard error, different lowercase letter (a, b, c, d) after the numbers indicate that the same index has significant differences among different treatments (*p* < 0.05).

**Table 4:** Physico-chemical properties of soybean rhizospheric soil under different cropping culture from Wuji country, Shijiazhuang city, China in 2023.

Sample ID	pH	SOC (g kg^−1^)	SOM (g kg^−1^)	TN (g kg^−1^)	AN (g kg^−1^)	NH_4_^+^-N (g kg^−1^)	NO_3_^−^-N (g kg^−1^)
MSN_0_	7.63 ± 0.13a	3.86 ± 0.12aA	6.66 ± 0.22aA	0.68 ± 0.07aA	28.56 ± 2.56aA	5.93 ± 0.23aA	11.42 ± 0.67daA
MSN_25_	7.53 ± 0.08ab	7.85 ± 0.19bA	13.53 ± 0.19b	1.08 ± 0.02bA	67.52 ± 3.18b	6.54 ± 0.31bA	12.03 ± 0.81aA
MSN_50_	7.61 ± 0.05a	7.38 ± 0.21bA	12.72 ± 0.45bA	1.11 ± 0.05b	81.83 ± 7.20c	6.02 ± 0.47abA	11.54 ± 0.93aA
MSN_100_	7.41 ± 0.12b	11.31 ± 0.37cA	19.50 ± 0.28cA	1.45 ± 0.02c	114.41 ± 7.53d	6.55 ± 0.55bA	24.83 ± 2.94bA
ISN_0_	7.67 ± 0.09a	6.44 ± 0.33aB	11.11 ± 0.32aB	0.79 ± 0.02aB	49.42 ± 2.54aB	7.16 ± 0.21bB	18.47 ± 1.24aB
ISN_25_	7.65 ± 0.14a	8.33 ± 0.21bB	14.37 ± 0.11b	0.90 ± 0.04bB	62.16 ± 2.69b	4.43 ± 0.28aB	37.28 ± 0.98 dB
ISN_50_	7.63 ± 0.04a	9.34 ± 0.45cB	16.10 ± 0.37cB	1.12 ± 0.03c	83.95 ± 4.32c	4.48 ± 0.17bB	22.12 ± 1.12bB
ISN_100_	7.41 ± 0.05b	12.44 ± 0.24 dB	21.45 ± 0.56 dB	1.43 ± 0.12d	113.57 ± 9.93d	5.43 ± 0.24bB	28.88 ± 2.37cB

MS, soybean in monocropping system; IS, soybean in MSSI system. The abbreviation N_0_, N_25_, N_50_, and N_100_ represent no N fertilizer, 75% N reduction, 50% N reduction and traditional N fertilizer, respectively. The abbreviation SOC, SOM, TN, AN, NH_4_^+^-N and NO_3_^−^-N represent soil organic carbon, soil organic matter, total N, available nitrogen, ammonium N and nitrate N, respectively. Data are mean ± SD (*n* = 3). Lowercase letters: differences among N levels in same cropping pattern (Tukey HSD test, *p* < 0.05); same letters = no significant difference. Uppercase letters: differences between Mono and MSSI groups at the same N level (independent samples *t*-test, *p* < 0.05); same letters = no significant difference.

### 3.5 Copy numbers of N-cycling genes in the soybean rhizosphere under the variable cropping system and N fertilization application

**Figure 4:**
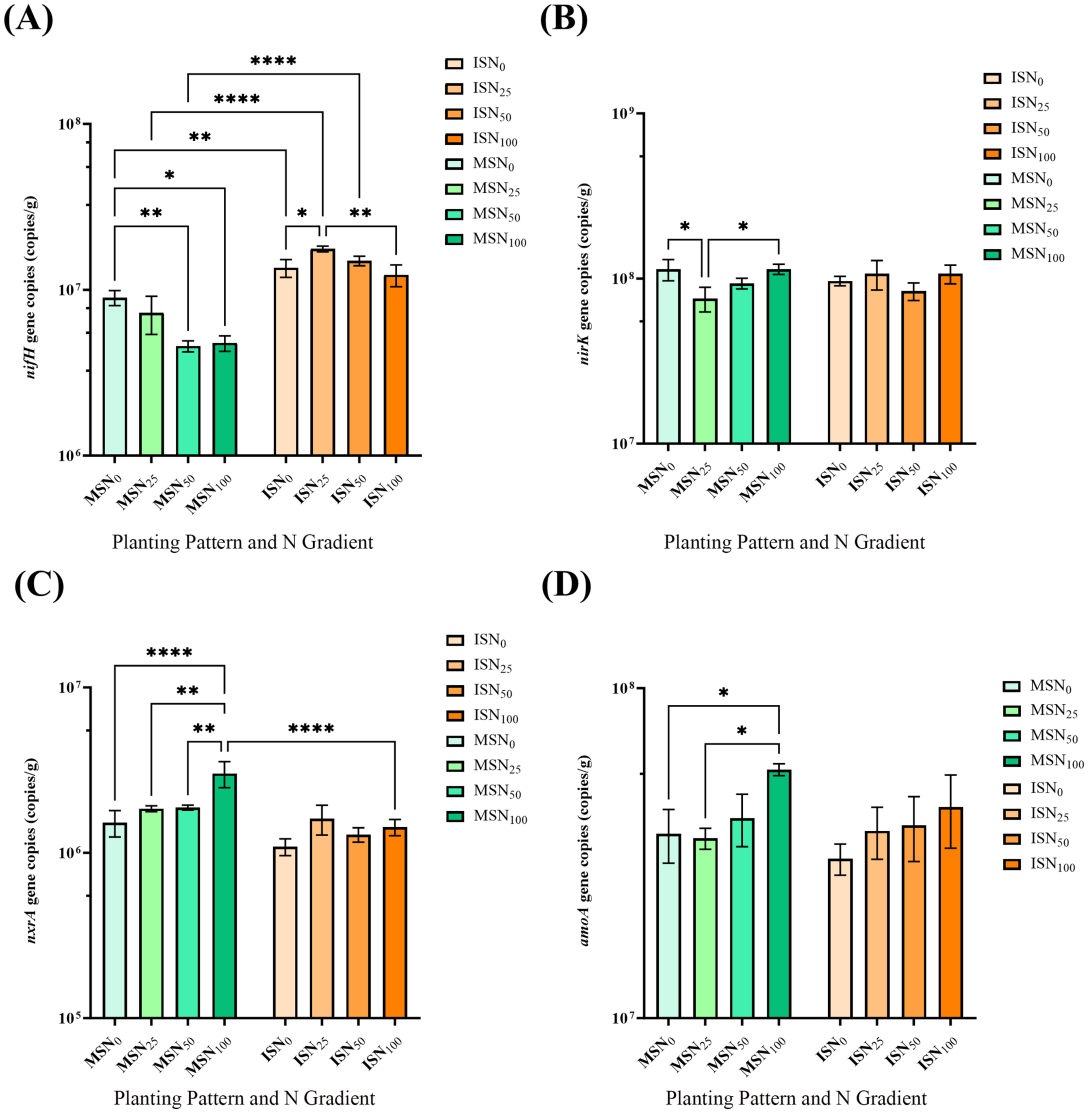
q-PCR detection of N fixation related genes in soybean rhizosphere soil. Quantitative detection of **(A)** *nifH*; **(B)** *nxrA*; **(C)** *amoA* and **(D)** *nirK*. IS, maize-soybean relay strip intercropping system. The abbreviation N_0_, N_25_, N_50_, and N_100_ represent no N fertilizer, 75% N reduction, 50% N reduction and traditional N fertilizer, respectively. Data are presented as means ± standard deviation (SD). ANOVA, Duncan test, * *p* < 0.05, ** *p* < 0.01, *** *p* < 0.001, **** *p* < 0.0001.

Rhizosphere soil samples from eight soybean communities were analyzed via RT-qPCR to quantify the abundance of N-cycling functional genes (Figure 4 and Supplementary Table S4).

For the *nifH* gene (a marker for N-fixing bacteria), the copy number followed the order: ISN_25_ > ISN_0_ > ISN_50_ > ISN_100_ > MSN_0_ > MSN_25_ > MSN_50_ > MSN_100_. Across all N fertilizer application levels, MSSI significantly increased *nifH* gene abundance compared with monocropping, with the highest value (1.76 × 10^7^ copies g^−1^) observed in ISN*_25_*—double that of MSN_25_ (Figure 4A). Notably, *nifH* abundance in monocropping decreased with increasing N input, while MSSI maintained high N-fixing bacterial counts even under N reduction. Two-way ANOVA confirmed that *nifH* abundance was significantly affected by cropping system, N rate, and their interaction (Supplementary Table S4), highlighting that MSSI enhances BNF to support efficient N use.

For nitrification-related genes (*nxrA* and *amo*A), their abundance in monocropping increased with N fertilizer application and were slightly higher than in MSSI overall (Figures 4C,D and Supplementary Table S4). In the N_0_ treatment, MSSI significantly reduced the abundance of these genes compared with monocropping, indicating suppressed nitrification under low N in the intercropping system.

Regarding the denitrification-related *nir*K gene (key for N loss via denitrification), no significant difference was detected between ISN_100_ and MSN_100_ (Figure 4B and Supplementary Table S4). However, MSSI showed a trend of reducing *nirK* abundance across most N levels, suggesting potential mitigation of N fertilizer loss from cropland ecosystems. These results demonstrate that MSSI reshapes the soybean rhizosphere N cycle by enhancing N-fixing bacterial abundance (via *nifH*) while suppressing nitrification and denitrification, with ISN_25_ being the optimal treatment to balance BNF and N retention.

### 3.6 Nitrogenase activity in rhizosphere soil under different cropping systems and N fertilization application

**Figure 5:**
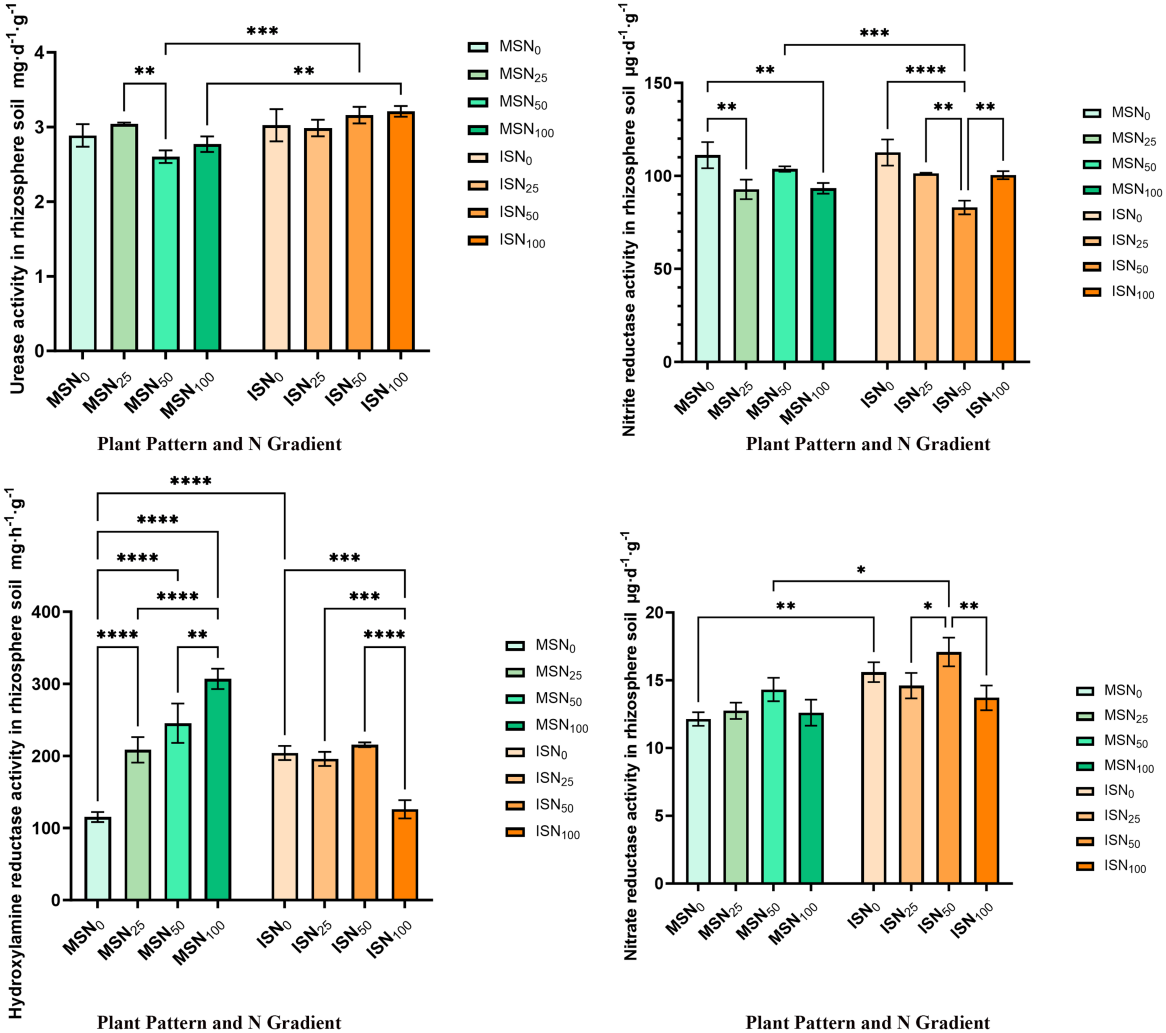
Quantitative determination of enzyme activities related to N cycle in soybean rhizosphere soil. Quantitative detection of **(A)** urease; **(B)** nitrite reductase; **(C)** hydroxylamine reductase and **(D)** nitrate reductase. IS, maize-soybean relay strip intercropping system. The abbreviation N_0_, N_25_, N_50_, and N_100_ represent no N fertilizer, 75% N reduction, 50% N reduction and traditional N fertilizer, respectively. Data are presented as means ± standard deviation (SD). ANOVA, Duncan test, * *p* < 0.05, ** *p* < 0.01, *** *p* < 0.001, **** *p* < 0.0001.

Urease activity is a key indicator reflecting soil N transformation potential, as it mediates urea hydrolysis to available N, directly linking to soil N supply capacity and fertility status. Two-way ANOVA results showed that urease activity was significantly regulated by N fertilizer application rates (*F* = 36.64, *p* = 0.0038) and the interaction between cropping pattern and N rate (*F* = 7.44, *p* = 0.0045), while cropping pattern alone had no significant effect (*F* = 224.1, *p* = 0.3330) (Supplementary Table S4). Notably, most MSSI treatments exhibited higher urease activity than monocropping, with no strong correlation to N fertilizer application levels (Figure 5A), indicating that intercropping modulates urease activity primarily through its interactive effect with N supply rather than independent regulation.

Hydroxylamine reductase, a critical enzyme in N cycling, was significantly affected by cropping pattern (*F* = 23.49, *p* = 0.0005), N rate (*F* = 55.87, *p* = 0.0017), and their interaction (*F* = 77.63, *p* < 0.0001) (Supplementary Table S4). In monocropping, hydroxylamine reductase activity increased with increasing N input (Figure 5C), whereas MSSI significantly suppressed this activity in the ISN_100_ treatment (*p* < 0.05). This suggests that MSSI mitigates the N-induced enhancement of hydroxylamine reductase activity under high N conditions, potentially regulating the rate of intermediate N transformation to avoid excessive N loss.

Nitrite reductase (NiR) catalyzes the reduction of NO₂^−^ and serves as a key enzyme in denitrification and inorganic N assimilation, with no involvement in nitrification (Stöhr and Ullrich, 2002). NiR activity was significantly influenced by cropping pattern (*F* = 21.78, *p* = 0.0022) and the interaction of cropping pattern × N rate (*F* = 14.69, *p* = 0.0003), whereas N rate alone had no significant effect (*F* = 0.2414, *p* = 0.6489) (Supplementary Table S4). Consistent with its positive correlation with crop yield, NiR activity in ISN_25_ was significantly higher than that in MSN_25_ (Figure 5B), indicating optimized N transformation potential under moderate N reduction in MSSI system.

Nitrate reductase (NR), which mediates the reduction of NO₃^−^ to NO₂^−^, acts as another core enzyme in denitrification and N assimilation and is also not involved in nitrification (Campbell and Kinghorn, 1990; Zhang et al., 2025). Unlike NiR, NR activity was significantly influenced by both cropping pattern (*F* = 9.557, *p* = 0.00111) and N rate (*F* = 61.86, *p* = 0.0014), with no significant interaction between the two factors (*F* = 2.083, *p* = 0.1560) (Supplementary Table S4). Across all treatments, MSSI consistently enhanced NR activity compared to monocropping (Figure 5D), reflecting the intercropping system’s ability to promote microbial nitrate utilization.

These results collectively demonstrate that MSSI optimizes rhizosphere N cycling by synergistically regulating the activities of key N-cycling enzymes: it enhances urease-mediated N mineralization (via the cropping pattern × N rate interaction), modulates hydroxylamine reductase to avoid intermediate N loss, and promotes NR/NiR activities to facilitate efficient inorganic N conversion. This enzymatic coordination ultimately improves soil N availability and plant N absorption capacity, which underpins the efficient N use efficiency (NUE) observed in MSSI system.

### 3.7 Impact of cropping system and N fertilization application on the diversity of rhizospheric microbial communities

Twenty-four soybean rhizosphere soil samples were studied. The rhizosphere soil samples were sequenced via the Illumina Miseq platform, and 783,700 valid sequences were obtained. A total of 2,300 OTUs were obtained via effective sequence clustering. The depth index of *nifH* gene sequencing ranged from 99.50–99.82% (Table 3). The quantity of sequence data acquired at this sequencing depth may provide the information on N-fixing bacteria in soybean rhizosphere soil samples.

To evaluate the alpha diversity of individual soil samples, the soil bacterial community richness (Chao1 index), diversity (Shannon index), and sequence depth (Goods_coverage) were determined for each N fertilizer treatment and cropping system (Table 3). The Chao1 index was used to measure species richness (i.e., larger values indicate the more species present in the community). Table 3 indicates that the Good’s_coverage for bacteria across all the treatments exceeded 99.5%. The Shannon and Simpson index indicates for bacterial community diversity showed similar variations among the treatments, with the maximum values occurring at MSN_100_ under both MS and IS plant cropping. Except for the N_25_ treatment, there was no obvious variation in rhizosphere soil diversity between the two cultivation modes at the same N fertilizer application level. Significant changes in the diversity of N-fixing bacteria were not detected when N application was reduced. The Chao1 index showed a similar pattern to that of Shannon diversity index in terms of bacterial community richness, as it was greater in ISN_25_ than in MSN_25_. The variety and richness of the N-fixing bacterial population peaked as additional N was lowered to the N_25_ level.

The PCoA of all 48 rhizosphere soil samples revealed that triplicate microbial communities within the same treatment clustered tightly but were distinctly separated from those in the other treatment, indicating high reproducibility among replicates. The samples from soybean were distributed far from the maize samples, suggesting great differences between the two soils (Supplementary Figure S1). The total variance explained by the different plant rhizosphere soil microbial communities (PCo1) was 70.4%, which suggested that the greater effect of the rhizosphere N-fixing bacteria in soybeans was significantly different from that in maize. The PCA plot also shows that MS and MM are significantly separated in the coordinate space, indicating substantial differences in crop physiological characteristics under monocropping systems. The intercropping treatments (IS and IM) were distributed between these two groups but clustered closer to the maize group (Supplementary Figure S1).

### 3.8 Community structure and composition of the *nifH*-based microbiota in the soybean rhizosphere

**Figure 6:**
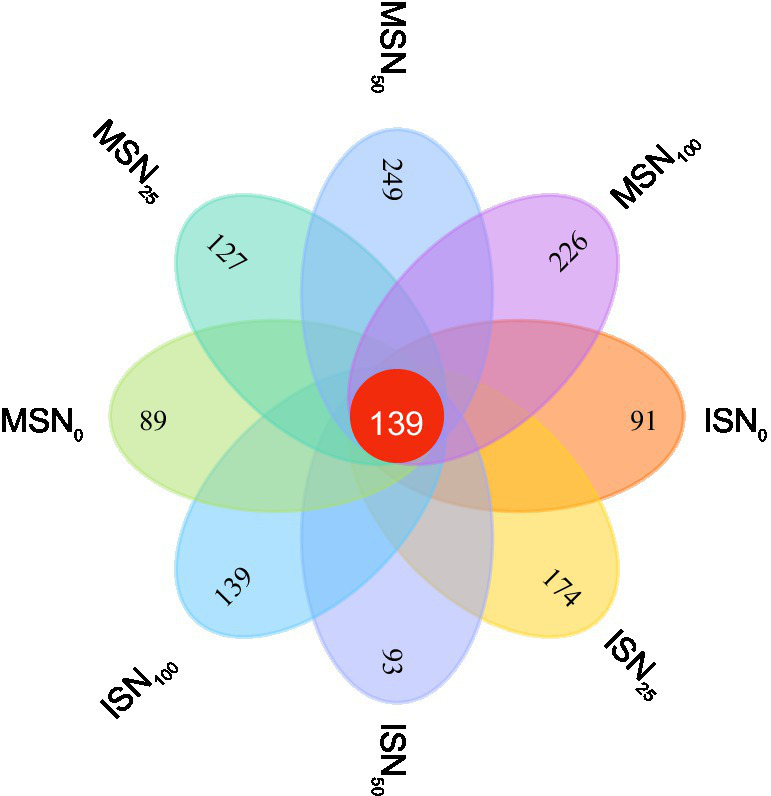
Venn diagrams of the OTU distribution under various planting patterns and N application levels.

A Venn diagram was used to compare the OTUs in the eight groups. A total of 11.7% (139) of the bacterial OTUs were shared among the eight experimental groups (Figure 6). The specific bacteria in each group accounted for 7.66% (91), 14.65% (174), 7.83% (93), 11.70% (139), 7.49% (89), 10.69% (127), 20.96% (249) and 19.23% (226) of the ISN_0_, ISN_25_, ISN_50_, ISN_100_, MSN_0_, MSN_25_, MSN_50_, and MSN_100_ OTUs, respectively. The results indicated that under zero N fertilization, the unique bacterial OTU composition was reduced in the soybean rhizosphere regardless of the planting pattern (monocropping or intercropping). Among the various treatments of MSSI treatments, ISN_25_ had a significantly greater unique bacterial OTU composition.

**Figure 7:**
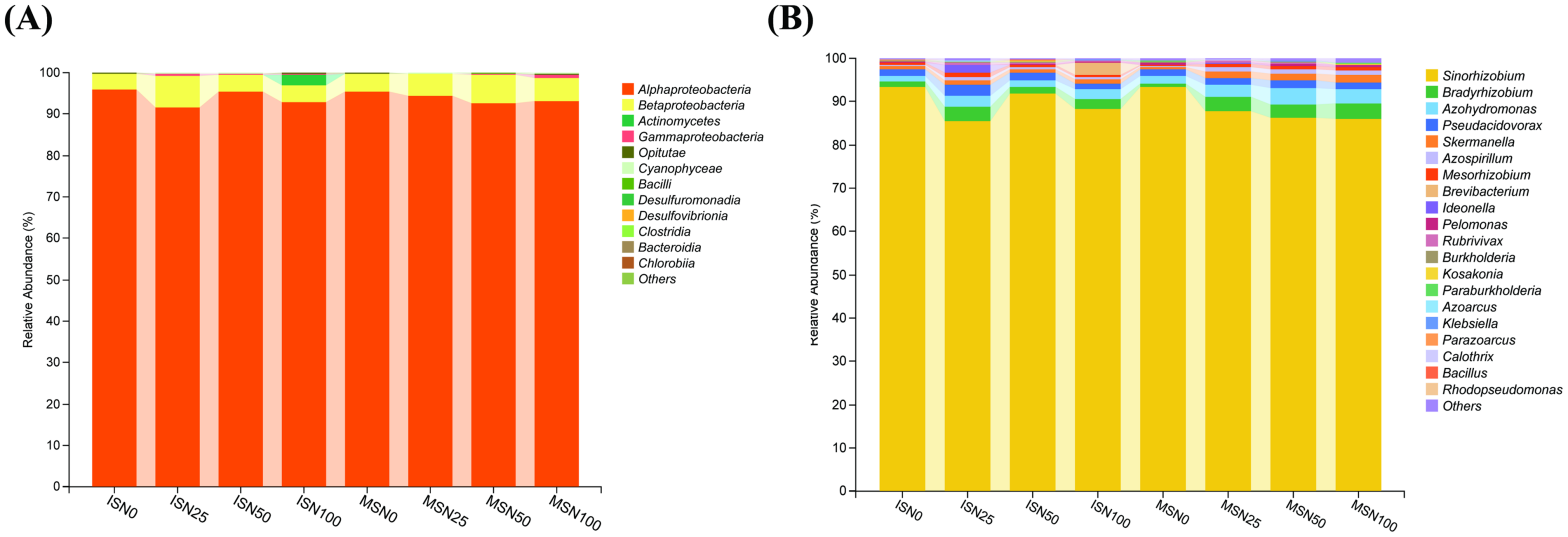
**(A)** Community composition of *nifH* gene at class level of soil N-fixing microorganisms under different treatments. **(B)** Community composition of *nifH* gene at genus level of soil N-fixing microorganisms under different treatments. IS, maize-soybean relay strip intercropping system. The abbreviation N_0_, N_25_, N_50_, and N_100_ represent no N fertilizer, 75% N reduction, 50% N reduction and traditional N fertilizer, respectively.

According to the species annotation of the sequencing results, a total of 8 phyla, 12 classes, 30 families and 37 genera of N-fixing bacteria were obtained from the 8 soybean rhizosphere soil samples (Figure 7). As shown in Figure 7A, a total of 12 classes of bacteria were detected, namely *Alphaproteobacteria*, *Betaproteobacteria*, *Actinomycetes*, *Gammaproteobacteria*, *Opitutae*, *Cyanophyceae*, *Bacilli*, *Desulfuromonadia*, *Desulfovibrionia*, *Clostridia*, *Bacteroidia* and *Chlorobiia*. Among the defined classes, *Alphaproteobacteria* was the dominant bacterial community in all the samples, with a relative abundance of 91.735 to 95.971%, and the highest abundance was found in the ISN_0_ plots. *Betaproteobacteria* is a subdominant class with a relative abundance of 3.692 to 7.501%. The communities of *Betaproteobacteria* in ISN_25_ were differed from those in the other plots, reaching up to 7.501%. The *Betaproteobacteria* community in the ISN_100_ plots was different from that in the other plots, with a relative abundance of 2.658%, which was much greater than in the other plots. At the genus level, the diversity of the ISN_25_ plots was significantly greater than that of the other plots. The dominant bacterial communities were *Sinorhizobium*, *Azohydromonas*, *Pseudacidovorax*, *Skermanella*, *Azospirillum*, *Mesorhizobium* and *Brevibacterium* (Figure 7B). Among the *genera* that can be defined, in every treatment, *Skermanella* was the predominant genus, with a relative abundance of 85.578 to 93.396%. *Bradyrhizobium* is the second dominant genus with a relative abundance of 0.929 to 3.559%.

### 3.9 Phylum- and genus-level heatmap profiling of *nifH*-based community structure differences in the soybean rhizosphere microbiota

**Figure 8:**
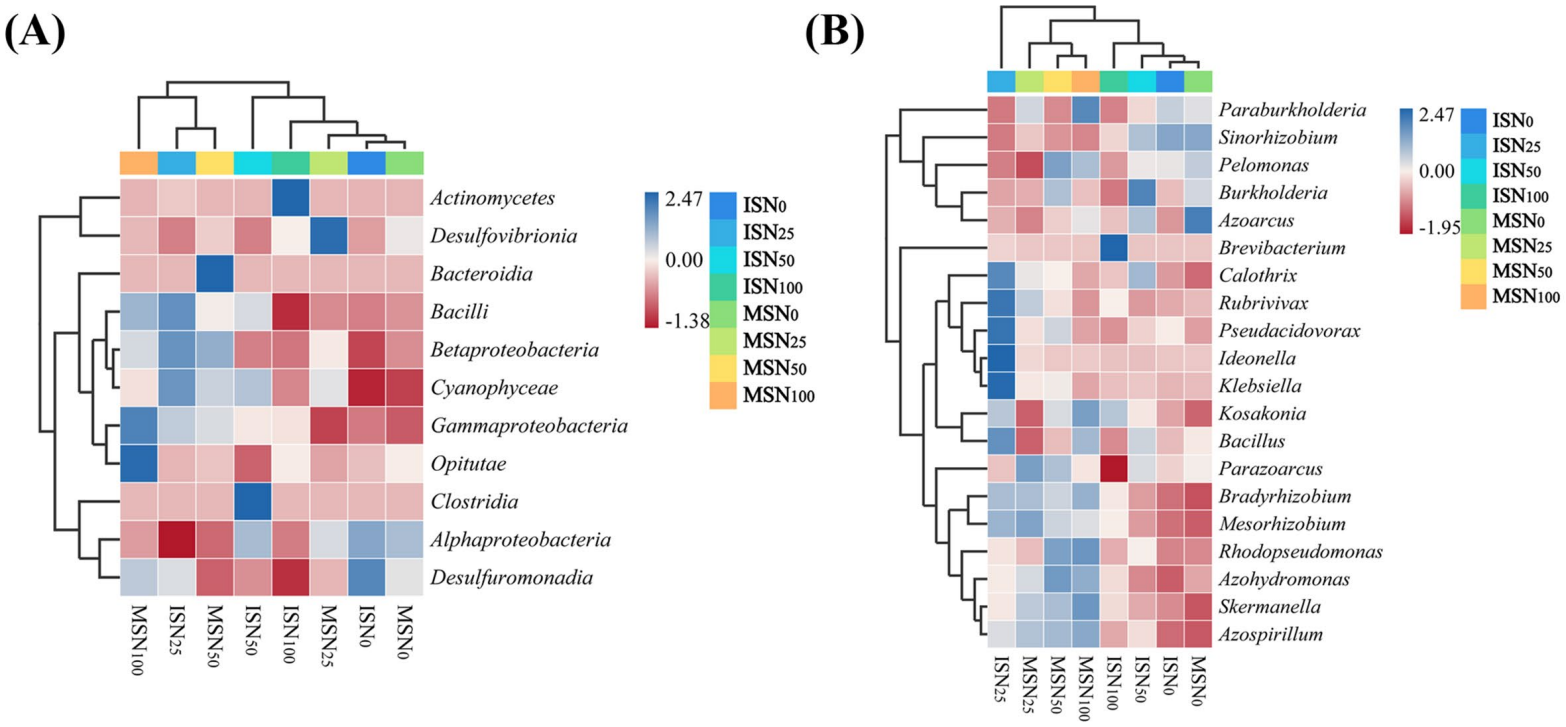
The heat map analysis of *nifH* gene-based community structure differences. **(A)** phylum-level heat map profiling of *nifH* gene-based community structure differences. **(B)** Genus-level heat map profiling of *nifH* gene-based community structure differences. Blue indicates a positive correlation, red coloring indicates a negative correlation.

Under various cropping patterns and N fertilizer application levels, the abundance of diazotrophic communities at the phylum level significantly differed. Figure 8A displays a heatmap illustrating the differential phyla within the soybean rhizosphere diazotrophs across treatments. The heatmap employs a color gradient from blue (high abundance) to red (low abundance), with values ranging from 1.38 to 2.47. The dominant phyla included *Actinobacteria*, *Deltaproteobacteria*, *Bacteroidia*, *Bacilli*, *Betaproteobacteria*, *Cyanophyceae*, etc. The results indicated that under the ISN_0_ treatment, certain phyla (e.g., *Deltaproteobacteria* and *Gammaproteobacteria*) appeared blue (high abundance). Conversely, under the MSN_100_ treatment, these taxa often shifted to red (low abundance), suggesting that the cropping pattern and N supply significantly regulate the phylum-level structure of rhizosphere diazotrophs.

Figure 8B focuses on the species level, highlighting differences in soybean rhizosphere bacteria under varying N levels. The differential species included *Paraburkholderia*, *Sinorhizobium* (a typical symbiotic N-fixing bacterium closely associated with soybeans), *Pseudomonas*, *Burkholderia*, *Azotobacter*, *Brevibacterium*, etc. Notably, known functional diazotrophs such as *Sinorhizobium*, *Bradyrhizobium*, *Azotobacter*, *Paenibacillus*, and *Azospirillum* showed abundance changes directly reflecting N-fixation potential. For example, under low N (ISN_0_), the abundance of these diazotrophs increased (blue), likely due to N stress-induced enhancement of N-fixation metabolism. Under high N (e.g., ISN_100_, MSN_100_), exogenous N suppressed N-fixation gene expression, leading to decreased abundance (red).

Distinct clustering patterns in diazotroph abundance were observed between the IS and MS treatments. These findings indicate that the cropping structure shapes the species-level community structure of N-fixing bacteria through alterations in the rhizosphere microenvironment (nutrient availability, root exudates, aeration, etc.).

### 3.10 Co-occurrence network analysis of *nifH* gene-based N-fixing bacterial communities

**Figure 9:**
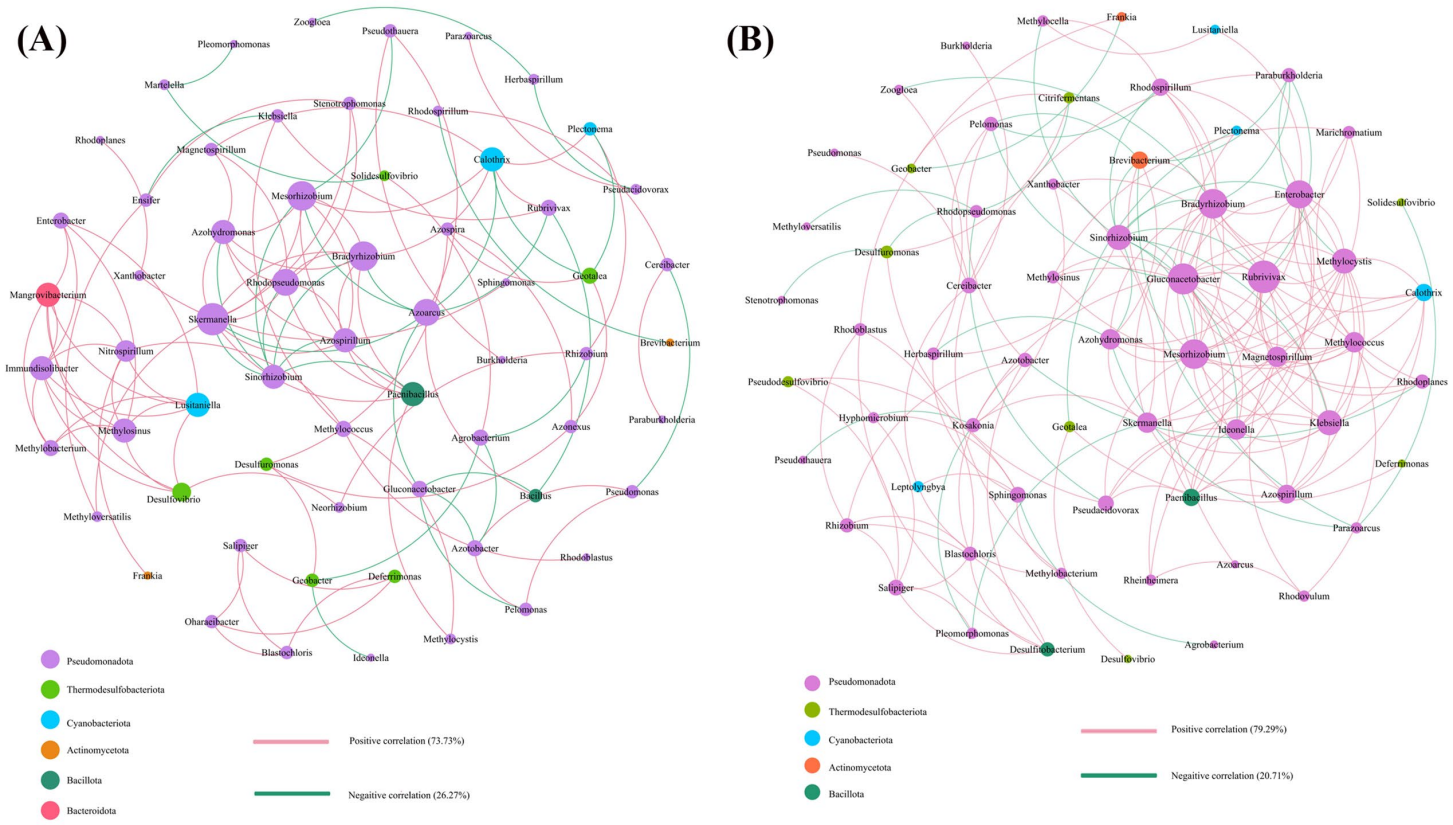
Co-occurrence network analysis of soil bacterial and fungal communities in the IS groups **(A)** and the MS groups **(B)**. The size of each node is proportional to the relative abundance of the genus. Each connection shown has a Spearman’s coefficient of >0.6 and *p*-value of <0.05. The red line shows a positive correlation, and the green line shows a negative correlation.

The symbiotic network interactions between the IS and MS groups were analyzed via Spearman’s correlation coefficient to reveal microbial co-occurrence patterns. Our findings indicate that bacteria play a central role in shaping the soil microbial symbiotic architecture, serving as the core functional components within these networks (Figure 9). Both the IS and MS groups contained rhizosphere N-fixing bacteria, dominated by the soybean specific symbiont *Bradyrhizobium*. In the IS group, the dominant bacterial taxa were *Gluconacetobacter*, *Rubrivivax*, and *Enterobacter*, whereas in the MS group, *Skermanella*, *Mesorhizobium*, and *Rhodopseudomonas* were the predominant classes, reflecting a notable shift in microbial community structure following microbially mediated soil intervention. These findings indicate that different planting patterns result in distinct core microbial members in each environment.

Interestingly, while the overall proportion of bacterial correlations slightly increased from 79.29% in the IS to 73.73% in the MS, the total number of interaction edges—representing the complexity and connectivity of the microbial network—increased from 42% in the MS group to 46.5% in the IS. This substantial increase suggests that the MSSI system not only altered the taxonomic composition of the dominant microbes but also enhanced the intricacy of their ecological interactions.

Notably, *Mangrovibacterium* exhibited increased correlation activity with other microbial members in the MS network, implying potential integration into more complex regulatory circuits. The high abundance of *Frankia* (actinobacterial N-fixer) in the IS network may inhibit pathogens by producing antimicrobial substances, whereas long-term continuous cropping in MS may lead to the accumulation of opportunistic pathogens such as *Enterobacter*, increasing the risk of soybean root rot. Collectively, these results suggest that the MSSI system effectively reshaped the soil microbiome by shifting dominance toward beneficial taxa and reconstructing interaction dynamics.

### 3.11 Pearson correlation analysis of *nifH* abundance, plant tissue N content and rhizosphere soil physicochemical properties

The copy number of the *nifH* gene in the soybean rhizosphere soil was compared with the soil physicochemical properties, the N content in the plant tissue and the enzyme activity of nitrogenase. The results of the Pearson correlation analysis revealed that (Table 5), the *nifH* gene copy number was significantly positively correlated with enzyme activity of soybean soil nitrite reductase and the N content in the seeds and leaves (*p* < 0.005). The correlation coefficients were 0.820, 0.670 and 0.654, respectively.

**Table 5:** Pearson correlation analysis between *nifH* gene copy number and soil physicochemical properties.

Pearson’s *P*-value	pH	SOC	SOM	TN	AN	NH_4_^+^-N	NO_3_^−^-N	Urease	NitrateRE	NitriteRE	HydroxylamineRE	N-leaf	N-seed
P	0.003	0.895	0.893	0.226	0.345	0.099	0.027	0.139	0.174	1.9 × 10^−7^	0.002	4.60 × 10^−7^	1.00 × 10^−12^
*nifH*	−0.416^*^	0.020	0.020	0.178	0.139	−0.241	−0.319^*^	−0.217	−0.199	0.670^**^	−0.431^*^	0.654^**^	0.820^***^

The abbreviation SOC, SOM, TN, AN, NH_4_^+^-N and NO_3_^−^-N represent soil organic carbon, soil organic matter, total N, available N, ammonium N and nitrate N, respectively. The abbreviations NitrateRE, NitriteRE, and HydroxylamineRE represent nitrate reductase, nitrite reductase and hydroxylamine reductase, respectively. MS, soybean in monocropping system; IS, soybean in MSSI system. The abbreviation N_0_, N_25_, N_50_, and N_100_ represent no N fertilizer, 75% N reduction, 50% N reduction and traditional N fertilizer, respectively. Pearson’s *p* -value, * *p* < 0.05, ** *p* < 0.01.

**Figure 10:**
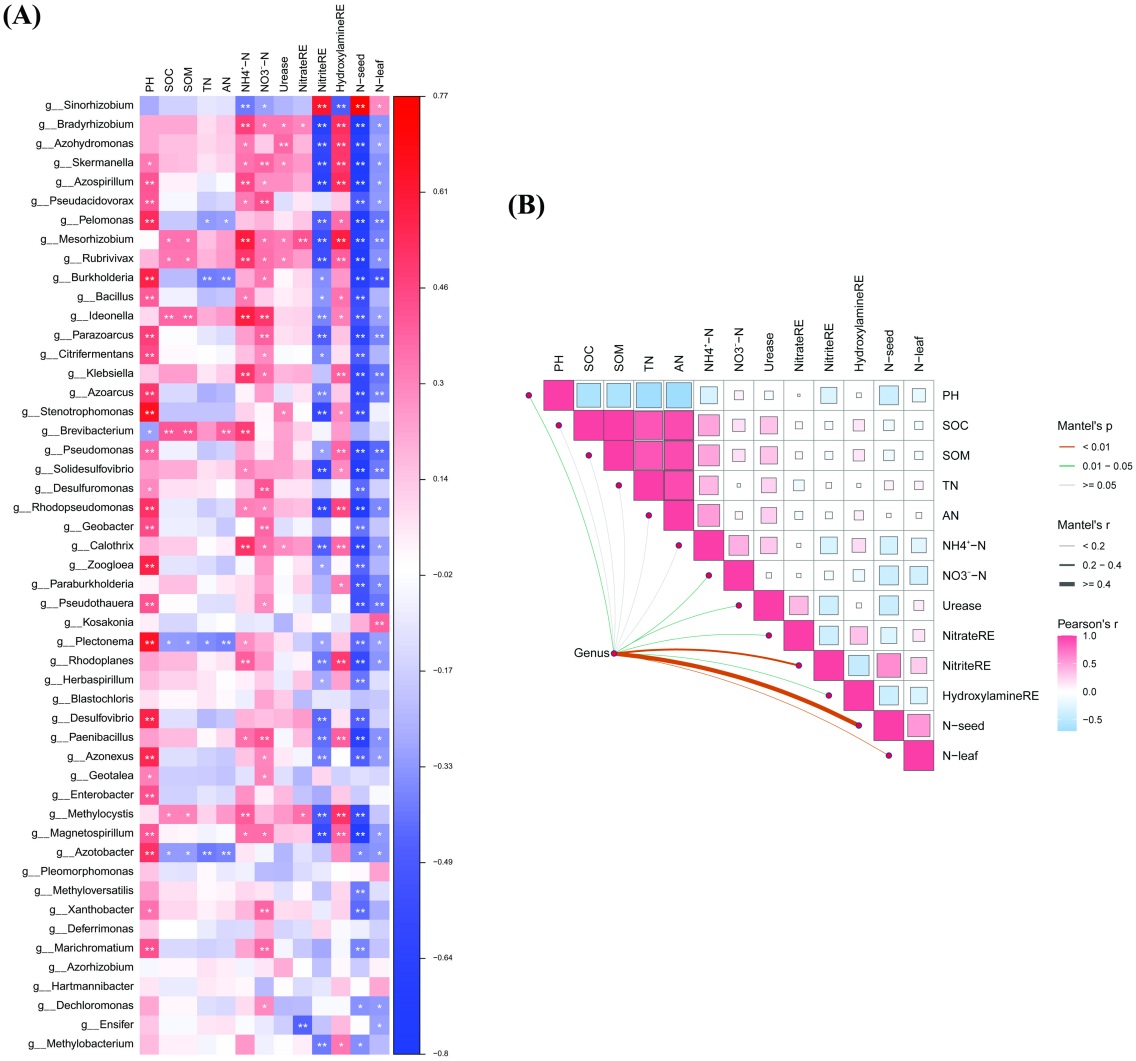
**(A)** Heatmap chart of correlation analysis between soil N-fix bacterial genera and soil physicochemical properties and N content in soybean. The correlation determined using Pearson analysis. Blue coloring indicates a positive correlation, orange coloring indicates a negative correlation, asterisks indicate a significant correlation. **(B)** Heatmap of Pearson correlations of soil dominant bacterial genera with soil factors, and N content in soybean. Cherry coloring indicates a positive correlation, Azure coloring indicates a negative correlation; the size of the squares and the darkness of color indicates correlation coefficient. ^*^p < 0.05 and ^**^p < 0.01.

The correlation heatmap can be visualized by the correlation number values, which show the relationships between different species and the environmental variables in the sample. Fifty N-fixing bacterial groups were correlated with soil physicochemical properties according to Spearman analysis. The results of the analysis are shown in Figure 9A. pH, TN, AN, and SOM and significantly affected the composition of the N-fixing bacterial community. The enzyme activity of nitrite reductase in the soil samples and the N content in the leaves significantly affected the N content in seeds. The N content in soybean seeds and the enzyme activity of nitrite reductase in soil samples significantly positively correlated with *g_Sinorhizobium.* The pH value significantly affected the N-fixing bacteria in the sample, such as *g_Skermanella*, *g_Azospirillum*, *g_Stenotrophomonas* and *g_Burkholderia*. AN and TN were significantly negatively correlated with *g_Pelomonas* and *g_Burkholderia*. SOM and SOC were significantly positively correlated with *g_Mesorhizobium*, *g_Rubrivivax*, *g_Ideonella*, and *g_Brevibacterium*. Soil N-fixation related enzyme activity significantly affects the colonization of N-fixing bacteria. NH_4_^+^-N and NO_3_^−^-N were significantly positively correlated with N-fixing bacteria. Nitrate reductase, urease and hydroxylamine reductase were significantly negatively correlated with *g_Bradyrhizobium*. A heatmap of the Pearson correlations (Figure 9B) revealed the positive correlations between enriched genera and pH, nitrite reductase, and N content in soybean leaves (see Figure 10).

## 4 Discussion

Soybean-maize strip intercropping (MSSI) systems optimize resource utilization through plant–soil-microbe interactions (Singh et al., 2026), achieve high-yield and green production primarily through efficient N acquisition, with soil N-fixing microorganisms playing a pivotal role in soil N cycling (Fan et al., 2020; Osakina and Jia, 2023; Xie et al., 2025). Long-term high N fertilization typically suppresses N-fixing microbial competitiveness and alters community structure (Guo et al., 2022; Yang et al., 2022), while N reduction often enhances their abundance and diversity. But the mechanistic links between cropping patterns, N management, and rhizospheric N-fixing communities remain poorly understood. This study elucidates how MSSI and reduced N fertilizer application co-regulate diazotrophic community assembly, soil N cycling processes, and crop productivity, while disentangling the microecological mechanisms underlying MSSI-induced improvements in system sustainability.

### 4.1 Ecological mechanisms driving *nifH* abundance-diversity divergence

The *nifH* gene serves as a marker for the potential size of the N-fixing bacterial pool, but its abundance does not directly equate to activity or efficiency (Li et al., 2023). A critical observation was that MSSI increased *nifH* abundance by 42–58% compared to monocropping (1.76 × 10^7^ copies g^−1^ in ISN_25,_ Figure 3A) but reduced Shannon and Simpson diversity (Table 4). This pattern reflected functional specialization driven by MSSI-mediated rhizosphere niche optimization (Li et al., 2022). MSSI modifies root exudates and alters soil C/N ratios, thereby selectively promoting symbiotic N-fixing bacteria-particularly *Sinorhizobium* (38% relative abundance in ISN_25_, Figure 5B), *Bradyrhizobium*, and *Azohydromonas* within the dominant *Pseudomonadota* phylum. These taxa adapted to interspecific interactions outcompete less competitive groups, driving increased proliferation of N-fixing populations while reducing community evenness. These specialized taxa outcompete less-adapted groups for resources, leading to increased *nifH* abundance (via population expansion of dominant genera) and reduced community diversity (via niche exclusion of non-adapted taxa) (Hu et al., 2026). This specialization aligns with increased soybean root nodule numbers in MSSI (Supplementary Table S4), as dominant taxa (e.g., *Ideonella* in ISN_25_) form effective symbiotic relationships with soybean roots, explaining why MSSI enhances N-fixing potential despite reduced diversity (Lai et al., 2022).

### 4.2 Synergistic effects of MSSI and reduced N

The ISN_25_ treatment (37.5 kg hm^−2^ N) emerged as the optimal N management strategy, increasing soybean yield by 18% while concurrently enhancing *nifH* gene abundance and nodulation (Table 2; Supplementary Table S5). Central to this optimization are two interconnected mechanisms, coupled with divergent responses of N-fixing communities across cropping systems.

First, low-N priming effect balancing early growth and nodulation. ISN_25_ stimulated early soybean root growth (47% higher root biomass than the N_0_ treatment) without suppressing nodule formation (Zaeem et al., 2021). This early root proliferation laid the foundation for subsequent nutrient acquisition and nodule development, addressing the critical N demand of soybean seedlings when root nodules are still underdeveloped and unable to supply sufficient N. As plants matured, enriched N-fixing communities (e.g., *Ensifer*, *Ideonella*) and increased nodules shifted the N supply from exogenous input to symbiotic fixation, ensuring reproductive-stage N availability (Zhu et al., 2024). This early root proliferation laid the foundation for subsequent nutrient acquisition and nodule development, addressing the critical N demand of soybean seedlings when root nodules are still underdeveloped and unable to supply sufficient N.

Second, MSSI mediated rhizosphere niche optimization. MSSI reshaped the root micro-environment by increasing SOC (15%) and NO_3_^−^-N (22%, Table 4), creating a resource-rich micro-environment that favors the colonization of functionally relevant N-fixing taxa. The enrichment of *Ideonella*-a marker taxon in ISN_25_ (Figure 5B)-confirms intercropping-induced niche differentiation. This differentiation serves as the foundation of divergent N-fixer responses between systems. In monocropping, N reduction suppressed *nifH* abundance and diversity. In contrast, MSSI system rhizosphere optimization enabled ISN_25_ to enhance these parameters.

In contrast, N reduction suppressed *nifH* abundance and diversity in monocropping, as it lacks capacity (like MSSI) to optimize rhizosphere resources. Without the complementary increase in SOC and balanced N availability, monocropping systems failed to support seedling growth or N-fixing microbial proliferation under reduced N inputs (Pang et al., 2022). Notably, high N inputs (N_50_, N_100_) consistently suppressed *nifH* abundance and nodule numbers in both systems, as excess exogenous N replaces biological N-fixation, reflecting the competitive exclusion of N-fixing bacteria under sufficient inorganic N supply (Cheng et al., 2023). Together, these results confirm that ISN_25_ strikes an optimal balance: it avoids N-induced suppression of N-fixing communities while meeting crop N demand across growth stages, leveraging both the priming effect of moderate N and MSSI’s niche optimization to enhance system sustainability (Shu et al., 2024).

### 4.3 Soil microenvironment and N-cycling enzymes

MSSI enriches symbiotic N-fixing bacteria by optimizing the rhizosphere microenvironment: negligible pH effects, increased SOC/SOM (fueling carbon demand), and enhanced N availability (e.g., nitrate N) under low N collectively create an optimal niche (Faust, 2021; Raza et al., 2020). N-cycling enzymes further reinforce this enrichment: higher urease releases labile N to meet copiotrophic demands; nitrite reductase activity (positively correlated with *nifH* gene abundance, *r* = 0.670) reduces NO_2_^−^ toxicity (a byproduct of nitrification) and supports denitrification flux; suppressed hydroxylamine reductase under high N minimizes N loss via nitrous oxide emisson and preserves soil N resources (Gao et al., 2021).

### 4.4 Mechanism of MSSI enhancing N-fixation via rhizosphere microenvironment and microbial synergy

MSSI and low-N fertilization application regulate N-fixing bacterial communities through a cascade of rhizosphere microenvironment and microbial-metabolic interactions, with no significant effect of soil pH (Table 4). The system enhances SOC accumulation by improving microbial metabolism and nutrient use balance, while boosting soil N supply (e.g., NO_3_^−^-N, NH_4_^+^- N in N_0_ treatment) via biological N-fixation and multi-pathway N cycle transfer (Raza et al., 2020). This nutrient enrichment selectively promotes dominant N-fixers: *Bradyrhizobium* (synergistic with *Rhizobium* for root N-fixation; Zhang et al., 2024) and *Ideonella* (enriched under ISN_25_), whose proliferation correlates with elevated urease, nitrite reductase activity (positively linked to *nifH* abundance, *r* = 0.670), and hydroxylamine reductase activity. Concurrently, MSSI low-N treatments suppress nitrate reductase activity and *amoA* gene abundance (a marker for ammonia-oxidizing potential), reducing ammonium N while promoting rhizosphere nitrate accumulation-likely driven by enhanced soybean root vitality and symbiotic N-fixing capacity, which collectively optimize N supply for crop demand and microbial niche differentiation.

## 5 Conclusion

This study found that cropping patterns and N levels reshape rhizospheric microenvironmental factors (e.g., C/N ratio, oxygen availability, host exudates) by regulating microbial community structure and functional taxon responses, thereby driving divergence in bacterial phylum abundance. High N reduces *Bacteroidia* abundance, potentially associated with suppressed lignin and cellulose degradation, while low N enriches Actinomycetes, linked to oligotrophic adaptation and auxiliary N-fixation. N-fixing functional groups (e.g., *Rhizobium*, *Bradyrhizobium*, *Azospirillum*) are sensitive to the interaction of cropping patterns and N gradients, with their dynamics governed by two ecological mechanisms: N deficiency enriches symbiotic and free-living N-fixers, whereas high N replaces biological N-fixation; MSSI optimizes the rhizospheric microenvironment to enhance N-fixer abundance at equivalent N levels.

This study confirmed that MSSI combined with N reduction treatment (ISN_25_, 37.5 kg hm^−2^) is the optimal scheme. It maximizes soybean yield while increasing nodule number, *nifH* gene abundance, and improving N use efficiency. This treatment fully exploits soybean’s symbiotic N fixation potential to meet N demand during the reproductive stage, providing a theoretical basis for microbiologically mediated green N fixation in soybean cultivation, reducing reliance on synthetic fertilizers, and illuminating the microbiome-driven mechanisms underlying the high productivity of intercropping systems.

## Data Availability

The data presented in this study are deposited in the NCBI Short Read Archive (SRA) with the accession number SRP678954, and all supporting experimental data (including physicochemical detection data and qPCR analysis results) are available in the Nutstore repository at the following link: https://www.jianguoyun.com/p/DXov42AQyd2GDhiLhJ0GIAA. All original contributions presented in the study are included in the article and its supplementary materials; further inquiries can be directed to the corresponding authors.

## Footnotes

1
https://docs.qiime2.org/2019.4/tutorials/
