# Supplementary material for: Effects of reduced nitrogen on the nifH-harboring soil microbiome in a soybean-maize strip intercropping system
Source: Front Microbiol. 2026 Mar 10;17:1770580. doi: 10.3389/fmicb.2026.1770580 (PMC13008894; doi:10.3389/fmicb.2026.1770580)
Supplement: Supplementary file 1 [file Data_Sheet_1.pdf]

Supplementary Material

Supplementary Figure 1

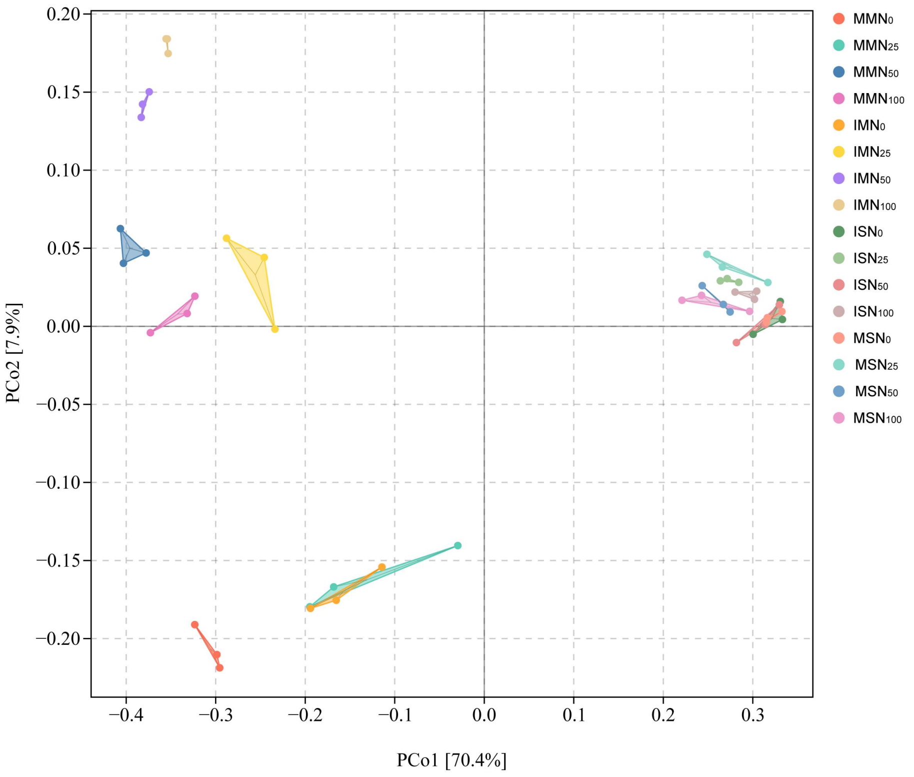

Supplementary Figure 1. PCoA analysis. MS, monoculture soybean; MSSI, maize-soybean relay strip intercropping system. IS, soybean in MSSI. MM, monoculture soybean. IM, maize in MSSI. The abbreviation N_0, N_{25}, N_{50}, and N_{100} represent no nitrogen fertilizer, 75% nitrogen reduction, 50% nitrogen reduction and traditional nitrogen fertilizer respectively.

Supplementary Tables

Supplementary Table 1. Experiment design

Supplementary Table 2. Primers used in this experiment

Supplementary Table 3. Three-way ANOVA analysis

Supplementary Table 4. Two-way ANOVA analysis

Supplementary Table 5. The nodules number per plant of soybean in different treatments

Supplementary Table 6. The crude protein and fat content of soybean in different treatments

Supplementary Table 1. Experiment design

ID	Cropping System	N-fertilizer Reduction	Crop
MMN$_{0}$	Mono	N$_{0}$ (No nitrogen fertilization)	Maize
MMN$_{25}$	Mono	N$_{25}$(Urea 37.5 $kg\cdot hm^{-2}$)	Maize
MMN$_{50}$	Mono	N$_{50}$(Urea 75.0 $kg\cdot hm^{-2}$)	Maize
MMN$_{100}$	Mono	N$_{100}$(Urea 150.0 $kg\cdot hm^{-2}$)	Maize
MSN$_{0}$	Mono	N$_{0}$ (No nitrogen fertilization)	Soybean
MSN$_{25}$	Mono	N$_{25}$(Urea 37.5 $kg\cdot hm^{-2}$)	Soybean
MSN$_{50}$	Mono	N$_{50}$(Urea 75.0 $kg\cdot hm^{-2}$)	Soybean
MSN$_{100}$	Mono	N$_{100}$(Urea 150.0 $kg\cdot hm^{-2}$)	Soybean
ISN$_{0}$	MSSI	N$_{0}$ (No nitrogen fertilization)	Soybean
ISN$_{25}$	MSSI	N$_{25}$(Urea 37.5 $kg\cdot hm^{-2}$)	Soybean
ISN$_{50}$	MSSI	N$_{50}$(Urea 75.0 $kg\cdot hm^{-2}$)	Soybean
ISN$_{100}$	MSSI	N$_{100}$(Urea 150.0 $kg\cdot hm^{-2}$)	Soybean
IMN$_{0}$	MSSI	N$_{0}$ (No nitrogen fertilization)	Maize
IMN$_{25}$	MSSI	N$_{25}$(Urea 37.5 $kg\cdot hm^{-2}$)	Maize
IMN$_{50}$	MSSI	N$_{50}$(Urea 75.0 $kg\cdot hm^{-2}$)	Maize
IMN$_{100}$	MSSI	N$_{100}$(Urea 150.0 $kg\cdot hm^{-2}$)	Maize

Supplementary Table 1. MS, monoculture soybean; MSSI, maize-soybean relay strip intercropping system. IS, soybean in MSSI. MM, monoculture soybean. IM, maize in MSSI. The abbreviation N$_{0}$, N$_{25}$, N$_{50}$, and N$_{100}$ represent no nitrogen fertilizer, 75% nitrogen reduction, 50% nitrogen reduction and traditional nitrogen fertilizer respectively.

Supplementary Table 2. Primers used in this experiment

Gene ID and reference	Primers	Sequence (5'to3')
amoA (Rotthauwe et al., 1997)	*amoA*-1F	GGGGTTTCTACTGGTGGT
	amoA-2R	CCCCTCKGSAAAGCCTTCTTC
nifH (Rösch et al.,2002)	*nifH*-F	AAAGGYGGWATCGGYAARTCCACCAC
	nifH-R	TTGTTSGCSGCRTACATSGCCATCAT
nxrA (Poly et al.,2008)	*nxrA*-F	CAGACCGACGTGTGCGAAAG
	nxrA-R	TCYACAAGGAACGGAAGGTC
nirK (Hallin et al.,1999)	*nir*KF1aCu	ATCATGGTSCTGCCGCG
	*nir*KR3Cu	GCCTCGATCAGRTTGTGGTT

Supplementary Table 2. Primers used in this experiment.

Supplementary Table 3. Three-way ANOVA Analysis

index	S(soil property)	Y(years)	C (Cropping pattern)	S×Y	S×C	C×Y	S×C×Y
nifH-soybean	F=6.402,P=0.1271,ns	F=0.7683,P=0.4732,*	F=48.97, P=0.0198,*	F=29.15, P=0.0.0326,*	F=4.23, P=0.1761,ns	F=0.500, P=0.5525,ns	F=185.4, P=0.0054,*
nifH-maize	F=813.6,P=0.0012,*	F=0.0188,P=0.9034,ns	F=53.87, P=0.0181,*	F=0.9376, P=0.4350,*	F=9.727, P=0.0809,ns	F=0.0755, P=0.8092,ns	F=0.3878, P=0.5970,ns
yield-soybean	F=47.66,P=0.0203,*	F=1764,P=0.0006,***	F=483.5, P=0.0021,**	F=142.2, P=0.0070,**	F=1895, P=0.0005,***	F=8034, P=0.0001,***	F=205.6, P=0.0048,**
yield-maize	F=4624,P=0.0002,***	F=164.6,P=0.0060,**	F=1.539, P=0.3406,ns	F=10.92, P=0.0807,ns	F=16.98, P=0.0541,ns	F=17.33, P=0.0521,ns	F=12.78 P=0.0701,*
height-soybean	F=26.25,P=0.0361,*	F=0.038,P=0.8635,ns	F=25508, P<0.0001,****	F=5.806, P=0.1376,ns	F=938.7, P=0.0011,**	F=194.1, P=0.0051,**	F=87.91, P=0.0112,*
height-maize	F=4561,P=0.0002,***	F=0.0005,P=0.9835,ns	F=23856, P<0.0001,****	F=44.76, P=0.0216,*	F=1175, P=0.0008,***	F=2272, P=0.0004,***	F=43.43, P=0.0223,*

Supplementary Table 3. The abbreviation S, Y,and C-Effect represent effect of soil property, year, cropping patten. P indicate significant differences, F represent degree of freedom. An asterisk (*) represents P<0.05, ** represents P<0.01, * represents P<0.001, **** represents P<0.0001， ns represents no significant differences.F represents F-value.**

Supplementary Table 4 Two-way ANOVA Analysis

Class	Index	N-Effect	C-Effect	N×C	N	C	N×C
Soil Physicochemical properties	pH	F=84.66, P<0.0001	F=0.77, P=0.4299	F=4.69, P=0.027	****	ns	*
	SOC	F=224.10, P<0.0001	F=99.62, P=0.0006	F=224.10, P<0.0001	****	***	****
	SOM	F=10776, P<0.0001	F=197.50, P=0.0001	F=300.70, P<0.0001	****	***	****
	TN	F=679.30, P<0.0001	F=0.64, P=0.4699	F=28.62, P<0.0001	****	ns	****
	AN	F=1133, P<0.0001	F=1.97, P=0.2335	F=37.18, P<0.0001	****	ns	****
	NH4-N	F=315.8, P<0.0001	F=22.16, P=0.0093	F=516.60, P<0.0001	****	**	****
	NO3-N	F=532.5, P<0.0001	F=199.8, P=0.0001	F=347.70, P<0.0001	****	***	****
Enzymatic Activity	Urease	F=224.1, P=0.3330	F=36.64, P=0.0038	F=7.44, P=0.0045	ns	**	**
	Nitrite reductase	F=21.78, P=0.0022	F=0.2414, P=0.6489	F=14.69, P=0.0003	**	ns	***
	Nitrate reductase	F=9.557, P=0.0111	F=61.86, P=0.0014	F=2.083, P=0.1560	*	**	ns
	Hydroxylamine reductase	F=23.49, P=0.0005	F=55.87, P=0.0017	F=77.63, P<0.0001	**	***	****
N-cycle genes	*nifH*	F=12.34, P=0.0041	F=207.4, P=0.0001	F=8.022, P=0.0034	**	***	**
	nxrA	F=12.02, P=0.0050	F=59.28, P=0.0015	F=7.374, P=0.0046	**	**	**
	nirK	F=2.945, P=0.1063	F=0.3112, P=0.6067	F=3.085, P=0.0681	ns	ns	ns
	amoA	F=6.412, P=0.0205	F=5.301, P=0.0827	F=1.175, P=0.3600	*	ns	ns
Plant N content	N-leaf	F=8.162, P=0.0147	F=904.6, P<0.0001	F=24.80, P<0.0001	*	****	****
	N-seed	F=14.32, P=0.0003	F=1.029, P=0.3677	F=11.04, P=0.0009	***	ns	***
	N-pod	F=8.194, P=0.0110	F=1.322, P=0.3144	F=0.01776, P=0.9966	*	ns	ns
Trait of Soybean	Height	F=59.16, P<0.0001	F=3.401, P=0.1389	F=252.9, P<0.0001	****	ns	****
	Seed num.	F=2210, P<0.0001	F=1.657, P=0.2674	F=587.7, P<0.0001	****	ns	****
	Yield	F=1385, P<0.0001	F=2436, P<0.0001	F=271.7, P<0.0001	****	****	****
	Nodules	F=653.4, P<0.0001	F=203.1, P=0.0001	F=24.53, P<0.0001	****	***	****
	Protein	F=126.3, P=0.0002	F=0.8685, P=0.4041	F=88.53, P<0.0001	***	ns	****
	Fat	F=273.7, P<0.0001	F=2.151, P=0.2163	F=19.82, P<0.0001	****	ns	****

Supplementary Table 4. The abbreviation N-Effect and C-Effect represent effect of N fertilizer application and effect of cropping patten respectively. F represent degree of freedom. P indicate significant differences, An asterisk (*) represents P<0.05, ** represents P<0.01, * represents P<0.001, **** represents P<0.0001, ns represents no significant differences.F represents F-value.**

Supplementary Table 5. The nodules number per plant of soybean in different treatments

Sample ID	Number of nodules
MSN_0	21.3±2.6aA
MSN_{25}	18.9±1.7bA
MSN_{50}	11.6±1.1cA
MSN_{100}	11.4±0.9cA
ISN_0	48.4±3.2aB
ISN_{25}	41.5±4.4bB
ISN_{50}	37.3±4.6bcB
ISN_{100}	34.6±3.4cB

Supplementary Table 5. MS, soybean in monocropping system; IS, soybean in MSSI system.The abbreviation N_0, N_{25}, N_{50}, and N_{100} represent no N fertilizer, 75% N reduction, 50% N reduction and traditional N fertilizer respectively. Data are mean ± SD (n=3). Lowercase letters: differences among N levels in same cropping patten (Tukey HSD test, P<0.05); same letters = no significant difference. Uppercase letters: differences between Mono and MSSI groups at the same N level (independent samples t-test, P<0.05); same letters = no significant difference.

Supplementary Table 6. The crude protein and fat content of soybean in different treatments

Sample ID	Protein (%)	Fat (%)
MSN_0	41.45±0.10	21.93±0.06a
MSN_{25}	41.89±0.07	21.45±0.03b
MSN_{50}	41.67±0.12	21.37±0.14b
MSN_{100}	41.54±0.11	21.89±0.09a
ISN_0	41.8.4±0.21	21.94±0.17
ISN_{25}	41.95±0.04	21.67±0.05
ISN_{50}	41.33±0.06	21.52±0.07
ISN_{100}	41.66±0.14	21.83±0.11

Supplementary Table 6. MS, soybean in monocropping system; IS, soybean in MSSI system.The abbreviation N_0, N_{25}, N_{50}, and N_{100} represent no N fertilizer, 75% N reduction, 50% N reduction and traditional N fertilizer respectively. Data are mean ± SD (n=3). Lowercase letters: differences among N levels in same cropping patten (Tukey HSD test, P<0.05); same letters = no significant difference. Uppercase letters: differences between Mono and MSSI groups at the same N level (independent samples t-test, P<0.05); same letters = no significant difference.
